# Reducing Systemic Inflammation in IUGR-Born Neonatal Lambs via Daily Oral ω-3 PUFA Supplement Improved Skeletal Muscle Glucose Metabolism, Glucose-Stimulated Insulin Secretion, and Blood Pressure

**DOI:** 10.3390/metabo15060346

**Published:** 2025-05-22

**Authors:** Melanie R. White, Rachel L. Gibbs, Pablo C. Grijalva, Zena M. Hicks, Haley N. Beer, Eileen S. Marks-Nelson, Dustin T. Yates

**Affiliations:** Stress Physiology Laboratory, Department of Animal Science, University of Nebraska-Lincoln, Lincoln, NE 68583, USA

**Keywords:** adaptive fetal programming, developmental origins of health and disease (DOHaD), glucose homeostasis, low birthweight, maternofetal health, omega-3 polyunsaturated fatty acid (ω-3 PUFA), placental insufficiency, small for gestational age (SGA)

## Abstract

Background/Objectives: Intrauterine growth restriction (IUGR) is associated with enhanced inflammatory activity, poor skeletal muscle glucose metabolism, and pancreatic β cell dysfunction that persist in offspring. We hypothesized that targeting heightened inflammation in IUGR-born neonatal lambs by supplementing anti-inflammatory ω-3 polyunsaturated fatty acids (ω-3 PUFAs) would improve metabolic outcomes. Methods: Maternal heat stress was used to produce IUGR lambs, which received daily oral boluses of ω-3 PUFA Ca^2+^ salts or placebo for 30 days. Results: Greater circulating TNFα and *semitendinosus* IL6R in IUGR lambs were fully resolved by ω-3 PUFA, and impaired glucose-stimulated insulin secretion, muscle glucose oxidation, and hypertension were partially rescued. Impaired glucose oxidation by IUGR muscle coincided with a greater glycogen content that was completely reversed by ω-3 PUFA and greater lactate production that was partially reversed. *Ex vivo* O_2_ consumption was increased in IUGR muscle, indicating compensatory lipid oxidation. This too was alleviated by ω-3 PUFA. Conversely, ω-3 PUFA had little effect on IUGR-induced changes in lipid flux and hematology parameters, did not resolve greater muscle TNFR1, and further reduced muscle β2-adrenoceptor content. Conclusions: These findings show that targeting elevated inflammatory activity in IUGR-born lambs in the early neonatal period improved metabolic outcomes, particularly muscle glucose metabolism and β cell function.

## 1. Introduction

Fetal programming associated with intrauterine growth restriction (IUGR) predisposes offspring to lifelong metabolic dysfunction [1,2], including impaired skeletal muscle glucose metabolism and poor insulin secretion [3,4]. Hypoxic stress from placental insufficiency is a primary driver of metabolic programming in IUGR fetal tissues [5,6,7,8], and the resulting hypercatecholaminemia was the earliest mechanism to be associated with metabolic dysfunction in the IUGR fetus [9,10,11]. However, fetal hypoxia also stimulates inflammatory cytokine secretion and upregulates their signaling pathways in muscle and other tissues, creating greater systemic inflammatory tone (i.e., greater systemic inflammation and tissue sensitivity to inflammatory factors) [12,13,14,15,16]. Recent studies have shown that heightened inflammatory tone persists in IUGR-born offspring despite the resolution of hypoxia and other stress factors at birth [17,18,19,20,21]. Inflammatory cytokines are potent disruptors of pancreatic islet function and skeletal muscle glucose metabolism [13,22,23,24]. In fact, experimental maternofetal inflammation produced a metabolic phenotype similar to that of other IUGR fetal models [13,25]. The ω-3 polyunsaturated fatty acids (ω-3 PUFAs) eicosapentaenoic acid (EPA; 20:5(*n*−3)) and docosahexaenoic acid (DHA; 22:6(*n*−3)) are highly anti-inflammatory [26,27,28,29]. Moreover, the infusion of EPA into IUGR fetuses for 5 days reduced circulating cytokines and improved muscle growth, myoblast function, and metabolic indicators near term [15,30]. Therefore, we hypothesized that the supplementation of ω-3 PUFA to IUGR-born neonatal lambs would likewise normalize inflammatory activity and improve deficits in metabolic health. Our objective was to test this hypothesis by evaluating indicators of systemic inflammation, pancreatic β cell function, and muscle-specific glucose metabolism in IUGR-born neonatal lambs following daily oral ω-3 PUFA supplementation from birth to 28 days of age.

## 2. Materials and Methods

### 2.1. Animals and Experimental Design

The research activities described in this manuscript were approved by the Institutional Animal Care and Use Committee at the University of Nebraska–Lincoln, which is accredited by AAALAC International. The study compared three groups of lambs: controls, IUGR-born lambs (IUGR), and IUGR-born lambs supplemented daily with ω-3 PUFA (IUGR+ω3). IUGR was produced as previously described [17,31]. Briefly, Polypay ewes were estrus-synchronized and timed-mated to a single sire. Randomly selected ewes were housed under hyperthermic conditions (40 ± 1 °C, 35 ± 5% relative humidity) from the 40th to the 95th day of gestation before being returned to thermoneutral conditions for the remainder of gestation. Control lambs (n = 12 from 7 ewes; 3 singletons, 6 twins, 3 triplets; 4 males, 8 females) were born to ewes that were pair-fed and maintained under thermoneutral conditions for the entirety of gestation. From birth, controls and IUGR lambs received daily oral boluses of molasses carrier only (IUGR; n = 11 from 10 ewes; 5 singletons, 4 twins, 2 triplets; 5 males, 6 females). IUGR+ω3 lambs received daily oral boluses of 0.420 g/kg Ca^2+^ salts of ω-3 PUFA (Strata, Virtus Nutrition, LLC, Corcoran, CA USA; n = 12 from 10 ewes; 3 singletons, 8 twins, 1 triplet; 5 males, 7 females) in 0.5 mL molasses. At birth, all lambs were weaned, given pooled colostrum, and hand-reared on *ad libitum* commercial milk replacer (Land O’ Lakes, Arden Hills, MN, USA). Blood samples were collected weekly via jugular venipuncture. At 23 days of age, heart rates and blood pressures were assessed via a multi-parameter veterinary monitor (Cardell Max 12-HD, Midmark, Dayton, OH, USA) as previously described [32]. Hindlimb catheters and blood flow probes were surgically placed on day 24, square-wave hyperglycemic clamps were performed on day 26, and hyperinsulinemic-euglycemic clamps (HEC) were performed on day 27, as previously described [31]. Lambs were euthanized via barbiturate overdose on day 28, and primary skeletal muscle was collected to evaluate ex vivo metabolism as previously described [24,31]. Power calculations (α = 0.05) indicated that 11 lambs/group was sufficient to detect 15% differences for in vivo glucose oxidation (0.91 power), ex vivo glucose oxidation (0.94 power), and O_2_ consumption rates (0.92 power), which are the outputs for which the greatest variation was expected.

### 2.2. Surgical Preparation

Lambs underwent surgical hindlimb catheterization under general anesthesia as previously described [31,33]. In brief, an indwelling Tygon catheter was placed just into the descending aorta via the femoral artery to allow for arterial blood sampling. In the contralateral hindlimb, a Precision S-Series Flow Probe (Transonic Systems, Inc., Ithaca, NY, USA) was placed around the femoral artery. The deep circumflex iliac artery and vein of this hindlimb were ligated and severed. Catheters were placed just into the inferior vena cava via the femoral vein in both hindlimbs for venous blood sampling and infusion. Catheters and flow probe cables were subcutaneously tunneled to the flank, exteriorized, and stored in a mesh pouch sutured to the skin.

### 2.3. In Vivo Metabolic Studies

#### 2.3.1. Square-Wave Hyperglycemic Clamps

To estimate insulin secretion under basal and hyperglycemic conditions, square-wave hyperglycemic clamps were performed as previously described [18,31]. In brief, lambs were placed in Panepinto slings and a series of 3 arterial blood samples was collected at 5-min intervals under resting conditions (i.e., basal). An intravenous bolus and infusion of 33% dextrose solution were then utilized to induce steady-state hyperglycemia at a target of 2.25-fold (±10%) greater blood glucose concentrations from the basal period. To measure second-phase insulin secretion, steady-state hyperglycemia was maintained for a minimum of 20 min before an additional series of 3 arterial blood draws was collected at 5-min intervals.

#### 2.3.2. Hyperinsulinemic–Euglycemic Clamps

Hindlimb-specific glucose metabolic rates were assessed during basal and hyperinsulinemic conditions, as previously described [19,31]. In brief, lambs were placed in Panepinto slings and infused with [^14^C(U)]-D-glucose (37.2 μCi/mL, PerkinElmer; Boston, MA, USA) at 2 mL/hour. After 40 min, four pairs of simultaneous arterial and venous blood samples were collected in 5-min intervals. Hyperinsulinemia was then induced by infusing insulin (250 mU/kg; Humulin R; Lilly, Indianapolis, IN, USA) at 4 mU/kg/minute. Lambs were maintained at euglycemia via variable-rate infusion of 33% dextrose. After 60 min of insulin infusion, four additional simultaneous arterial and venous blood samples were collected at 5-min intervals. Blood flow rates were recorded at the time of each blood sample. The Fick principle was used to estimate hindlimb-specific metabolic flux [31,34], and hindlimb glucose utilization and oxidation were quantified from differences in the concentrations of glucose and radiolabeled ^14^CO_2_ between arterial and venous samples. Differences were normalized to blood flow rate at sampling and to hindlimb weight at necropsy. Due to catheter failures in two lambs, HEC studies were performed in 12 controls, 11 IUGR lambs, and 10 IUGR+ω3 lambs.

### 2.4. Blood Analyses

Heparinized whole blood was used to determine circulating glucose, lactate, partial pressures of CO_2_ (pCO_2_) and O_2_ (pO_2_), oxyhemoglobin (O_2_Hb), carboxyhemoglobin (COHb), HCO_3_^−^, base excess, hemoglobin, hematocrit, Na^+^, K^+^, Cl^−^, and Ca^2+^ with an ABL90 FLEX blood gas analyzer (Radiometer, Brea, CA, USA) as previously described [31]. EDTA-treated whole blood was used to quantify total white blood cells, lymphocytes, monocytes, granulocytes, hematocrit, hemoglobin, red blood cells, platelets, mean red blood cell volumes, mean corpuscular hemoglobin concentrations, red blood cell distribution widths, and mean platelet volumes with a HemaTrue Veterinary Hematology Analyzer (Heska, Loveland, CO, USA), as previously described [31,35,36]. Blood counts were used to calculate the aggregate systemic inflammation index (AISI; (neutrophils × platelets × monocytes)/lymphocytes) [37], systemic immune inflammation index (SII; (neutrophils × platelets)/lymphocytes) [38], and systemic inflammatory response index (SIRI; (neutrophils × monocytes)/lymphocytes) [39]. The remaining EDTA-treated whole blood was centrifuged at 14,000× *g* for 5 min to isolate plasma, which was stored at −80 °C. Blood plasma urea nitrogen (BUN), total triglycerides, and high-density lipoprotein-bound cholesterol (HDLC) were measured on a Vitros 250 Analyzer (Ortho Diagnostics, Raritan, NJ, USA) by the Biomedical and Obesity Research Core at the University of Nebraska. Blood plasma insulin, non-esterified fatty acids (NEFA), and tumor necrosis factor-α (TNFα) concentrations were quantified in duplicate using commercial ELISA kits (Bovine Insulin, Alpco Diagnostics, Windham, NH, USA; NEFA, Wako Life Sciences, Richmond, VA, USA; Ovine TNFα, Fine Test, Wuhan, China) as previously described [15,17,32]. Inter-assay and intra-assay coefficients of variance were less than 15% for all assays.

### 2.5. Ex Vivo Skeletal Muscle Metabolism

#### 2.5.1. Glucose Uptake and Oxidation

Intact longitudinal strips of the *flexor digitorum superficialis* (FDS) muscle were used to determine muscle-specific glucose uptake and oxidation rates as described previously [24,31]. Muscle strips were incubated in Krebs-Henseleit bicarbonate buffer containing 0.1% bovine serum albumin (KHB) and spiked with 5 mM D-glucose (MilliporeSigma, Burlington, MA, USA) and 0 or 5 mU/mL insulin for 1 h at 37 °C and 5% CO_2_. Muscle strips were then washed in glucose-free KHB with the respective insulin concentration for 20 min at 37 °C and 5% CO_2_. To estimate glucose uptake, muscle strips (38.4 ± 4.0 mg) were incubated in KHB spiked with 1 mM [^3^H] 2-deoxyglucose (300 µCi/mmol; PerkinElmer), 39 mM [1-^14^C] mannitol (1.25 µCi/mmol; PerkinElmer), and the respective insulin concentration for 20 min at 37 °C and 5% CO_2_. Muscle strips were lysed using 2M NaOH, and specific activities for ^3^H and ^14^C were determined via liquid scintillation to estimate glucose uptake (i.e., intracellular 2-deoxyglucose accumulation) and intracellular fluid volume, respectively. To estimate glucose oxidation, a separate set of muscle strips (69.6 ± 3.8 mg) was incubated in sealed dual-well chambers in O_2_-saturated KHB spiked with 5 mM [^14^C-U]-D-glucose (0.25 µCi/mmol) and 0 or 5 mU/mL insulin for 2 h at 37 °C. Radiolabeled CO_2_ produced by the muscle strip and liberated from media via HCl was captured by NaOH in the adjoining well, and liquid scintillation was used to measure ^14^C specific activity. Specific activities of the known amounts of radiolabeled 2-deoxyglucose and glucose in the media were used to calculate glucose uptake and oxidation as nmol, which were normalized to muscle strip mass and time in incubation.

#### 2.5.2. Oxygen Consumption Rates

O_2_ consumption rates were used to indicate the total oxidative metabolic activity of intact longitudinal FDS muscle strips via Oxytherm + oxygen chambers (Hansatech Instruments, Ltd., King’s Lynn, UK). Muscle strips (84.9 ± 12.2 mg) were incubated in KHB spiked with 5 mM D-glucose and 0 or 5 mU/mL insulin. Media were equilibrated to atmospheric conditions at 37 °C for 1 min just prior to starting data collection. The air-tight chambers were then sealed, and O_2_ content in the media was quantified via a charged electrode every 0.1 s for 15 min at 37 °C with mild agitation from a glass stir bar, per manufacturer instructions. O_2_ consumption rates were averaged over the stabilized slope. Basal and insulin-spiked conditions were assessed in each of two chambers (i.e., two technical reps/media condition for each lamb).

### 2.6. Skeletal Muscle Protein Expression

Total protein was isolated from *semitendinosus* muscle that was frozen in liquid nitrogen at necropsy, as previously described [17,18]. Muscle samples were homogenized via sonication (3 × 5 s) in a low-salt TRIS-NaCl buffer +2.5% protease +2.5% phosphatase inhibitor and then centrifuged (14,000× *g*, 5 min, 4 °C). Supernatant was collected and total protein was quantified using a Pierce BCA Assay Kit (Thermo Fisher, Waltham, MA, USA). Then, 50-μg protein aliquots were mixed with Bio-Rad 4× Laemmli sample buffer (Bio-Rad Laboratories, Inc., Hercules, CA, USA) and heated at 95 °C for 5 min. Samples were equilibrated to room temperature and separated by SDS-PAGE prior to transfer to Bio-Rad poly-vinylidene fluoride low-fluorescent membranes. Membranes were washed with tris-buffered saline with tween (TBS-T) and incubated with Bio-Rad EveryBlot Blocking Buffer at room temperature for 10 min. Membranes were then incubated at 4 °C overnight with rabbit anti-serum raised against tumor necrosis factor receptor-1 (TNFR1; 1:500, C25C1, Cell Signaling Technologies, Danvers, MA, USA), rabbit anti-serum raised against interleukin-6 receptor (IL6R; 1:1000, clone EPR24322-143, Abcam Ltd., Cambridge, UK), rabbit anti-serum raised against toll-like receptor 4 (TLR4; 1:500, Ag13841, Proteintech, Rosemont, IL, USA), or rabbit anti-serum raised against β_2_-adrenergic receptor (β_2_-AR; 1:500, c-term, Cohesion Biosciences, London, UK). Finally, membranes were washed in TBS-T and incubated at room temperature for 1 h with goat anti-rabbit IR800 IgG secondary anti-serum (LI-COR Biosciences, Lincoln, NE, USA). Membranes were scanned using a LI-COR Odyssey Infrared System, and protein bands were analyzed with Image StudioLite Software 5.2. Each protein of interest was normalized to total protein.

### 2.7. Muscle Glycogen Content

Samples from the mid-portion of the *semitendinosus, longissimus dorsi*, and *biceps femoris* muscles were collected at necropsy, snap-frozen, and stored at −80 °C. Intramuscular glycogen content was quantified as previously described [17]. In brief, 50 mg of muscle was sonicated in double-distilled water for 20 s, heated at 95 °C for 5 min, and centrifuged at 14,000× *g* for 5 min. Duplicate 5-μL aliquots of supernatant were used to quantify glycogen content with a commercial colorimetric kit (MAK016; MilliporeSigma). Intra-assay and inter-assay coefficients of variance were less than 15%

### 2.8. Statistical Analysis

Data collected at single time points were analyzed as a linear mixed model by ANOVA using the mixed procedure of SAS 9.4 (SAS Institute, Cary, NC, USA) for the fixed effects of experimental group, sex, and birth number, as previously described [17,31]. Fisher’s LSD test was used for mean separation. Data for weekly blood components, in vivo metabolic studies, and ex vivo studies were analyzed using the mixed procedure with repeated measures to analyze the effects of experimental group, age/study period/incubation condition, and the interaction, as well as sex and birth number. Best-fit statistics were used to select the most appropriate covariance structures. Numbers for each sex and birth number category are reported in the methods. As reported in previous studies with a similar design, lamb was considered the experimental unit [17,31]. Significant differences for all analyses were identified by a *p*-value of ≤0.05, and tendencies were indicated by *p*-values of ≤0.10. All data are presented as ls means ± standard errors.

## 3. Results

### 3.1. Weekly Blood Parameters

#### 3.1.1. Plasma ω-3 PUFA and TNFα

**Figure 1:**
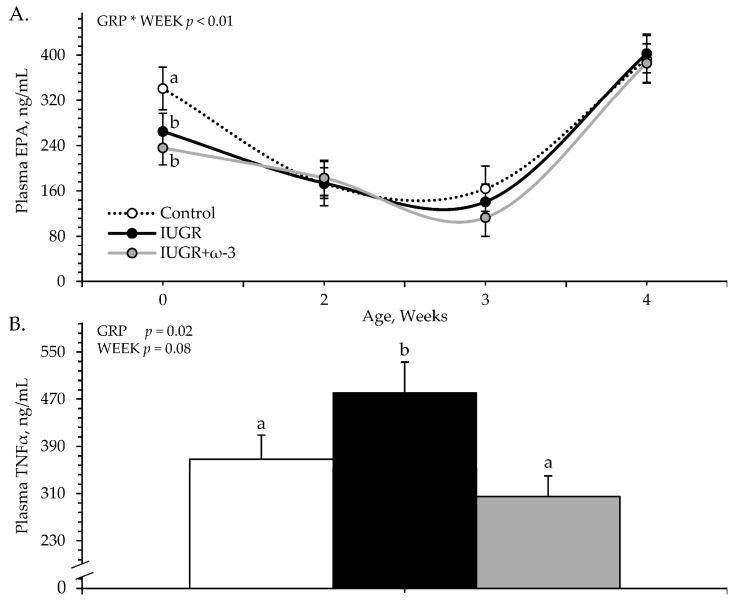
Circulating eicosapentaenoic acid (EPA) (**A**) and tumor necrosis factor-α (TNFα) (**B**) concentrations in IUGR-born neonatal lambs administered daily oral ω-3 PUFA supplements. Weekly blood samples were collected in control (n = 12), IUGR (n = 11), and IUGR+ω-3 lambs (n = 11). Effects of experimental group (GRP), week of age (WEEK), and their interaction (GRP × WEEK) were evaluated and are noted where significant (*p* < 0.05). ^a,b^ Means with different superscripts differ (*p* < 0.05).

An experimental group × age interaction was observed (*p* < 0.05) for plasma EPA but not DHA or TNFα concentrations. Plasma EPA was less (*p* < 0.05) for IUGR and IUGR+ω3 lambs than for controls at birth but did not differ among groups at any other age (Figure 1A). Plasma DHA did not differ among controls (42.0 ± 9.4 pg/mL), IUGR lambs (31.3 ± 7.9 pg/mL), and IUGR+ω3 lambs (42.1 ± 9.8 pg/mL). Plasma TNFα was greater (*p* < 0.05) for IUGR lambs but not IUGR+ω3 lambs than for controls (Figure 1B) and tended to decrease (*p* = 0.08) with age.

#### 3.1.2. Weekly Blood Gases and Metabolites

No experimental group × age interactions were observed for any weekly blood gases or metabolites. Regardless of age, blood glucose concentrations did not differ among control (5.6 ± 0.1 mM), IUGR (5.8 ± 0.1 mM), or IUGR+ω3 (5.6 ± 0.1 mM) lambs. Blood lactate concentrations were greater (*p* < 0.05) for IUGR (1.90 ± 0.15 mM) and IUGR+ω3 (1.91 ± 0.16 mM) lambs than for controls (1.51 ± 0.11 mM). Blood pH (7.408 ± 0.006), pO_2_ (44.1 ± 2.1 mmHg), pCO_2_ (49.4 ± 0.8 mmHg), HCO_3_ (30.3 ± 0.4 mM), base excess (5.6 ± 0.4 mM), hemoglobin (11.79 ± 0.17 g/dL), hematocrit (36.1 ± 0.5%), and oxyhemoglobin (68.2 ± 1.8%) did not differ among experimental groups. Blood carboxyhemoglobin tended to be greater (*p* = 0.10) for IUGR (2.50 ± 0.17%) but not IUGR+ω3 (2.33 ± 0.17%) lambs than for controls (2.03 ± 0.16%).

#### 3.1.3. Weekly Blood Electrolytes

No experimental group × age interactions were observed for any weekly blood electrolytes. Regardless of age, blood Na^+^ (159.8 ± 0.9 mM), K^+^ (5.11 ± 0.07 mM), and Cl^−^ (116.6 ± 0.8 mM) concentrations did not differ among experimental groups. Blood Ca^2+^ concentrations were greater (*p* < 0.05) for IUGR (1.37 ± 0.01 mM) and IUGR+ω3 (1.39 ± 0.02 mM) lambs than for controls (1.33 ± 0.01 mM).

#### 3.1.4. Leukocytes and Hematology

**Figure 2:**
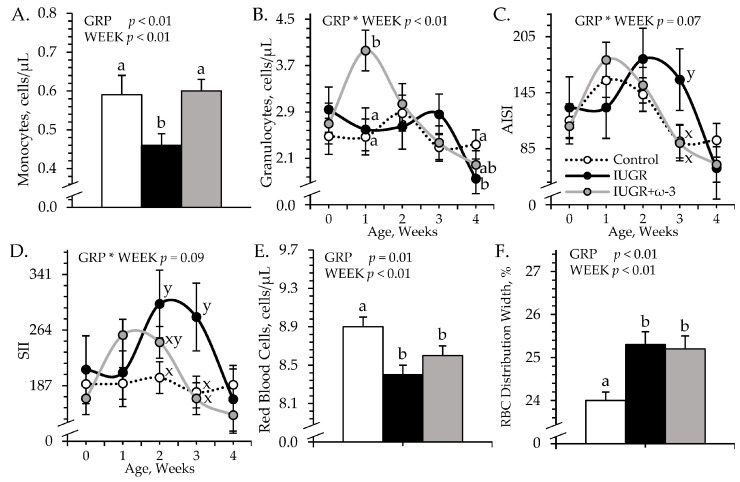
Blood cell metrics in IUGR-born neonatal lambs administered daily oral ω-3 PUFA supplements. Weekly blood samples were collected in control (n = 12), IUGR (n = 11), and IUGR+ω-3 lambs (n = 11). Data are shown for circulating monocytes (**A**), circulating granulocytes (**B**), aggregate systemic inflammation index (AISI) (**C**), systemic immune inflammation index (SII) (**D**), circulating red blood cells (**E**), and red blood cell distribution width (**F**). Effects of experimental group (GRP), week of age (WEEK), and their interaction (GRP × WEEK) were evaluated and are noted where significant (*p* < 0.05). ^a,b^ Means with different superscripts differ (*p* < 0.05). ^x,y^ Means with different superscripts tend to differ (*p* < 0.10).

An experimental group × age interaction was observed (*p* < 0.05) for blood granulocytes, AISI, and SII, but not for any other leukocyte or hematology component. Regardless of age, total white blood cells (6.14 ± 0.25 cells/μL) and blood lymphocyte concentrations (2.96 ± 0.11 cells/μL) did not differ among experimental groups. Blood monocytes were less (*p* < 0.05) for IUGR but not IUGR+ω3 lambs than for controls (Figure 2A). Blood granulocytes were greater (*p* < 0.05) for IUGR+ω3 lambs but not IUGR lambs than for controls at 7 days of age and were less (*p* < 0.05) for IUGR lambs but not for IUGR+ω3 lambs than for controls at 28 days of age (Figure 2B). AISI and SII tended to be greater (*p* < 0.09) for IUGR lambs but not IUGR+ω3 lambs than for controls at 21 days of age (Figure 2C,D). SII also tended to be greater (*p* = 0.09) for IUGR but not IUGR+ω3 lambs than for controls at 21 days of age. SIRI (0.53 ± 0.04) did not differ among groups. Blood hematocrit (32.7 ± 1.5%), hemoglobin (11.4 ± 0.1 g/dL), and mean corpuscular hemoglobin concentration (36.6 ± 0.1 g/dL) did not differ among experimental groups. Total red blood cells were less (*p* < 0.05) and red blood cell distribution width was greater (*p* < 0.05) for IUGR and IUGR+ω3 lambs than for controls (Figure 2E,F). Mean corpuscular volume was greater (*p* < 0.05) for IUGR (36.6 ± 0.2 fL) and IUGR+ω3 (37.0 ± 0.3 fL) lambs than for controls (35.4 ± 0.1 fL), regardless of age. Mean platelet volume tended to be greater (*p* = 0.08) for IUGR (5.05 ± 0.03 fL) but not IUGR+ω3 (4.96 ± 0.03 fL) lambs than for controls (4.97 ± 0.02 fL). Blood platelets (221.1 ± 7.1 per μL) did not differ among experimental groups.

### 3.2. Cardiovascular Parameters

**Figure 3:**
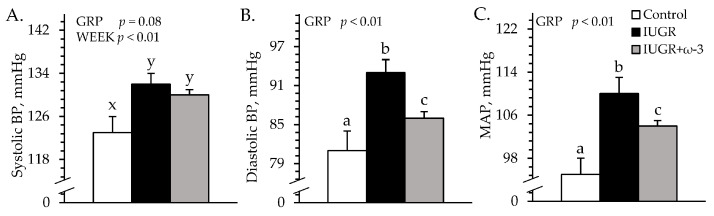
Blood pressure in IUGR-born neonatal lambs administered daily oral ω-3 PUFA supplements. Forelimb blood pressure was measured in control (n = 12), IUGR (n = 11), and IUGR+ω-3 lambs (n = 11). Data are shown for systolic (**A**), diastolic (**B**), and mean arterial blood pressure (MAP) (**C**). Effects of experimental group (GRP) were evaluated and are noted where significant (*p* < 0.05). ^a,b,c^ Means with different superscripts differ (*p* < 0.05). ^x,y^ Means with different superscripts tend to differ (*p* < 0.10).

Heart rates (171 ± 10 bpm) did not differ among experimental groups. Systolic blood pressure tended to be greater (*p* = 0.08) for IUGR and IUGR+ω3 lambs than for controls (Figure 3A). Diastolic blood pressure and mean arterial pressure were greater (*p* < 0.05) for IUGR lambs than controls and were intermediate for IUGR+ω3 lambs (Figure 3B,C).

### 3.3. Square-Wave Hyperglycemic Clamps

#### 3.3.1. Blood Gases and Metabolites

**Figure 4:**
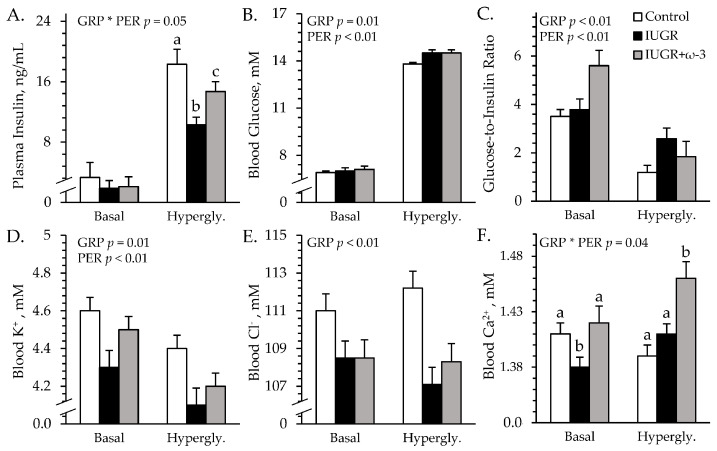
Glucose-stimulated insulin secretion in IUGR-born neonatal lambs administered daily oral ω-3 PUFA supplements. Blood was sampled under basal conditions and during a square-wave hyperglycemic clamp in control (n = 12), IUGR (n = 11), and IUGR+ω-3 lambs (n = 11). Data are shown for circulating insulin (**A**), glucose (**B**), glucose-to-insulin ratios (**C**), K^+^ (**D**), Cl^−^ (**E**), and Ca^2+^ (**F**). Effects of experimental group (GRP), period (PER), and their interaction (GRP × PER) were evaluated and are noted where significant (*p* < 0.05). ^a,b,c^ Means with different superscripts differ (*p* < 0.05).

Experimental group × study period interactions were observed (*p* < 0.05) for plasma insulin concentrations but not for any other parameters measured during hyperglycemic clamps. Basal plasma insulin did not differ among groups (Figure 4A). At hyperglycemia, plasma insulin was less (*p* < 0.05) for IUGR lambs than controls and was intermediate for IUGR+ω3 lambs. Regardless of the period, blood glucose and glucose-to-insulin ratios were greater (*p* < 0.05) for IUGR and IUGR+ω3 lambs than for controls (Figure 4B,C). Glucose-to-insulin ratios were also less (*p* < 0.05) during hyperglycemia than at basal conditions. Plasma NEFA (0.90 ± 0.07 mEq/L), total triglycerides (41.0 ± 3.3 mg/dL), and HDL-C (40.8 ± 0.9 mg/dL) concentrations did not differ among groups or periods. BUN concentrations were greater (*p* < 0.05) for IUGR+ω3 lambs (11.3 ± 0.2 mg/dL) than controls (9.4 ± 0.3 mg/dL) or IUGR lambs (9.4 ± 0.2 mg/dL). Blood lactate concentrations (0.71 ± 0.04 mM) were not different among groups. Blood pH was greater (*p* < 0.05) for IUGR and IUGR+ω3 lambs than for controls (Figure S1A). Blood pO_2_ (74.6 ± 0.8 mmHg) did not differ among groups (Figure S1B), but blood pCO_2_ was greater (*p* < 0.05) for IUGR lambs than for controls and was greatest (*p* < 0.05) for IUGR+ω3 lambs (Figure S1C). Blood HCO_3_^−^ and base excess were greater (*p* < 0.05) for IUGR and IUGR+ω3 lambs than for controls (Figure S1D,E). Hemoglobin concentrations were less (*p* < 0.05) for IUGR lambs and greater (*p* < 0.05) for IUGR+ω3 lambs than for controls (Figure S1F). Hematocrit was less (*p* < 0.05) for IUGR lambs and greater (*p* < 0.05) for IUGR+ω3 lambs than for controls (Figure S1G). Oxyhemoglobin was greater (*p* < 0.05) for IUGR and IUGR+ω3 lambs than for controls (Figure S1H). Carboxyhemoglobin was greater (*p* < 0.05) for IUGR lambs than for controls and was intermediate for IUGR+ω3 lambs (Figure S1I).

#### 3.3.2. Blood Electrolytes

An experimental group × study period interaction was observed (*p* < 0.05) for blood Ca^2+^ concentrations, but not for any other electrolytes measured during the hyperglycemic clamps. Regardless of the period, blood Na^+^ (148.1 ± 0.6 mM) did not differ among groups. Blood K^+^ was less (*p* < 0.05) for IUGR lambs but not for IUGR+ω3 lambs than for controls (Figure 4D). Blood Cl^-^ was less (*p* < 0.05) for IUGR and IUGR+ω3 lambs than for controls (Figure 4E). Basal blood Ca^2+^ was less (*p* < 0.05) for IUGR lambs but not for IUGR+ω3 lambs than for controls (Figure 4F). At hyperglycemia, blood Ca^2+^ was greater (*p* < 0.05) for IUGR+ω3 but not IUGR lambs than for controls.

### 3.4. Hyperinsulinemic–Euglycemic Clamps

#### 3.4.1. Hindlimb Glucose Metabolism

**Figure 5:**
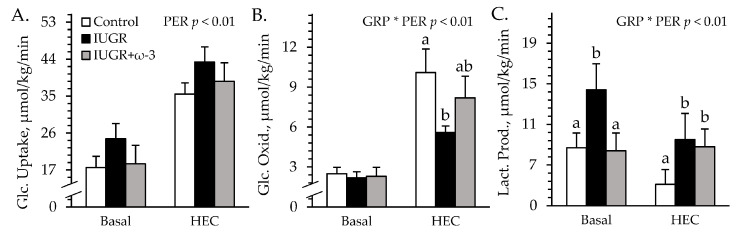
Hindlimb glucose metabolism in IUGR-born neonatal lambs administered daily oral ω-3 PUFA supplements. Blood was sampled under resting conditions (basal) and during a hyperinsulinemic-euglycemic clamp in control (n = 12), IUGR (n = 11), and IUGR+ω-3 lambs (n = 10). Data are shown for rates of glucose uptake (**A**), glucose oxidation (**B**), and lactate production (**C**). Effects of experimental group (GRP), study period (PER), and their interaction (GRP × PER) were evaluated and are noted where significant (*p* < 0.05). ^a,b^ Means with different superscripts differ (*p* < 0.05).

Experimental group × study period interactions were observed (*p* < 0.05) for hindlimb glucose oxidation and lactate secretion, but not for hindlimb glucose uptake during the HEC studies. Glucose uptake rates did not differ among groups under basal or HEC conditions (Figure 5A). Basal glucose oxidation rates did not differ among groups (Figure 5B). Under HEC conditions, glucose oxidation was less (*p* < 0.05) for IUGR lambs but not for IUGR+ω3 lambs than for controls. Basal lactate secretion was greater (*p* < 0.05) for IUGR lambs but not for IUGR+ω3 lambs than for controls (Figure 5C). Under HEC conditions, lactate secretion was greater (*p* < 0.05) for IUGR and IUGR+ω3 lambs than for controls. Blood flow rates (68 ± 5 L/minute) did not differ among groups or between periods.

#### 3.4.2. Blood Gases and Metabolites

**Figure 6:**
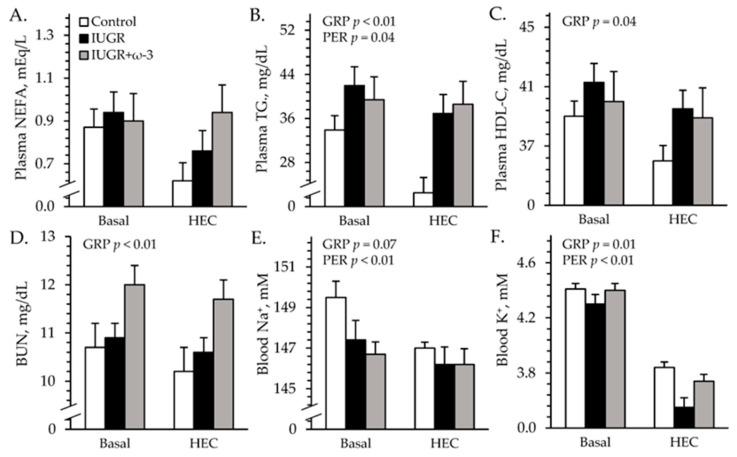
Metabolic parameters in IUGR-born neonatal lambs administered daily oral ω-3 PUFA supplements. Blood was sampled under resting conditions (basal) and during a hyperinsulinemiceuglycemic clamp in control (n = 12), IUGR (n = 11), and IUGR+ω-3 lambs (n = 10). Data are shown for plasma non-esterified fatty acids (NEFA) (**A**), plasma triglycerides (TG) (**B**), plasma HDL-bound cholesterol (HDL-C) (**C**), blood plasma urea nitrogen (BUN) (**D**), blood Na^+^ (**E**), and blood K^+^ (**F**). Effects of experimental group (GRP), study period (PER), and their interaction were evaluated and are noted where significant (*p* < 0.05).

Experimental group × study period interactions were observed (*p* < 0.05) for blood pH and base excess, but not for any other parameters measured during the HEC studies. Plasma insulin did not differ among groups but was greater (*p* < 0.05) under HEC conditions (27.1 ± 0.7 ng/mL) than at basal conditions (1.8 ± 0.6 ng/mL) by study design. Regardless of the period, blood glucose was greater (*p* < 0.05) for IUGR lambs (6.3 ± 0.1 mM) but not for IUGR+ω3 (6.3 ± 0.1 mM) lambs than for controls (6.3 ± 0.1 mM). Blood lactate was greater (*p* < 0.05) for IUGR (0.56 ± 0.02 mM) and IUGR+ω3 (0.56 ± 0.02 mM) lambs than for controls (0.50 ± 0.02 mM). Plasma NEFA did not differ among groups or between study periods (Figure 6A). Total blood triglycerides were greater (*p* < 0.05) for IUGR and IUGR+ω3 lambs than for controls (Figure 6B). Blood HDL-C was greater (*p* < 0.05) for IUGR lambs but not for IUGR+ω3 lambs than for controls (Figure 6C). BUN was greater (*p* < 0.05) for IUGR+ω3 lambs but not for IUGR lambs than for controls (Figure 6D). Blood pH did not differ among groups at basal conditions but was greater (*p* < 0.05) for IUGR and IUGR+ω3 lambs than for controls during HEC conditions (Figure S2A). Blood pO_2_ was less (*p* < 0.05) and pCO_2_ was greater (*p* < 0.05) for IUGR lambs but not for IUGR+ω3 lambs than for controls (Figure S2B,C). Blood HCO_3_^−^ was greater (*p* < 0.05) for IUGR lambs than for controls and was intermediate for IUGR+ω3 lambs (Figure S2D). Blood base excess did not differ among groups at basal conditions but was greater (*p* < 0.05) for IUGR and IUGR+ω3 lambs than for controls under HEC conditions (Figure S2E). Hemoglobin and hematocrit concentrations were less (*p* < 0.05) for IUGR lambs and greater (*p* < 0.05) for IUGR+ω3 lambs than for controls (Figure S2F,G). Oxyhemoglobin did not differ among groups or between periods, but carboxyhemoglobin was greater (*p* < 0.05) for IUGR lambs but not for IUGR+ω3 lambs than for controls (Figure S2H,I).

#### 3.4.3. Blood Electrolytes

No experimental group × period interactions were observed for electrolytes during the HEC study. Regardless of the period, blood Na^+^ tended to be less (*p* = 0.07) for IUGR and IUGR+ω3 lambs than for controls (Figure 6E). Blood K^+^ was less (*p* < 0.05) for IUGR lambs but not IUGR+ω3 than for controls (Figure 6F). Blood Cl^-^ was less (*p* < 0.05) for IUGR (107.2 ± 0.8 mM) and IUGR+ω3 (108.5 ± 0.4 mM) lambs than controls (110.6 ± 0.4 mM). Blood Ca^2+^ (1.40 ± 0.01 mM) did not differ among groups.

### 3.5. Ex Vivo Glucose Metabolism

**Figure 7:**
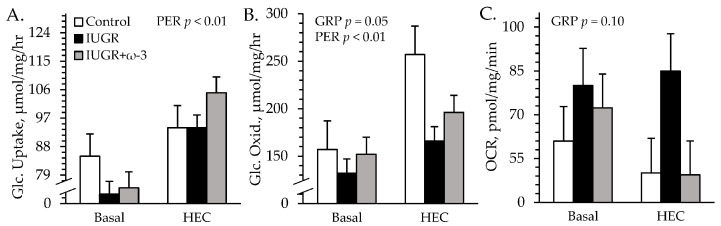
*Ex vivo* glucose metabolism in IUGR-born neonatal lambs administered daily oral ω-3 PUFA supplements. Primary *flexor digitorum superficialis* muscle from control (n = 12), IUGR (n = 11), and IUGR+ω-3 lambs (n = 11) was incubated with 0 (basal) or 5 mU/mL insulin. Data are shown for glucose uptake rate (**A**), glucose oxidation rate (**B**), and total O_2_ consumption rate (OCR) (**C**). Effects of experimental group (GRP), study period (PER), and their interaction were evaluated and are noted where significant (*p* < 0.05).

No experimental group × media interactions were observed for ex vivo skeletal muscle uptake or oxidation. Glucose uptake rates did not differ among groups but were greater (*p* < 0.05) in insulin-spiked media than in basal media (Figure 7A). Glucose oxidation rates were less (*p* < 0.05) for muscle from IUGR lambs but not IUGR+ω3 lambs than from control lambs, regardless of media (Figure 7B). Glucose oxidation rates were also greater (*p* < 0.05) in insulin-spiked media (207 ± 13 μmol/mg/hour) than in basal media (147 ± 13 μmol/mg/hour). Oxygen consumption rates tended to be greater (*p* = 0.10) for muscle from IUGR lambs but not IUGR+ω3 lambs than from controls (Figure 7C).

### 3.6. Muscle Protein and Glycogen Content

**Figure 8:**
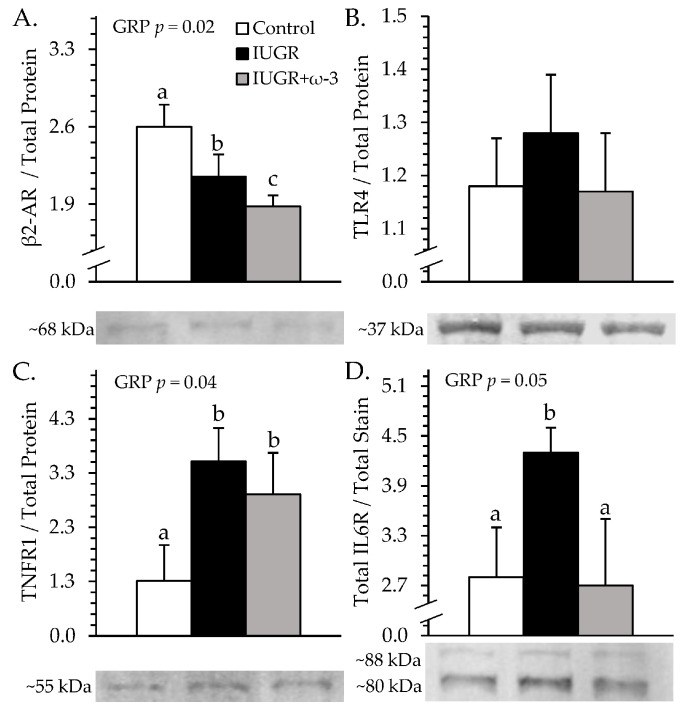
Skeletal muscle receptor content in IUGR-born neonatal lambs administered daily oral ω-3 PUFA supplements. *Semitendinosus* protein isolates were assessed for control (n = 12), IUGR (n = 11), and IUGR+ω-3 lambs (n = 11). Data are shown for β2 adrenoceptor (β2-AR) (**A**), TLR4 (**B**), TNFR1 (**C**), and IL6R (**D**) protein content. Effects of experimental group (GRP) were evaluated and are noted where significant (*p* < 0.05). ^a,b,c^ Means with different superscripts differ (*p* < 0.05).

**Figure 9:**
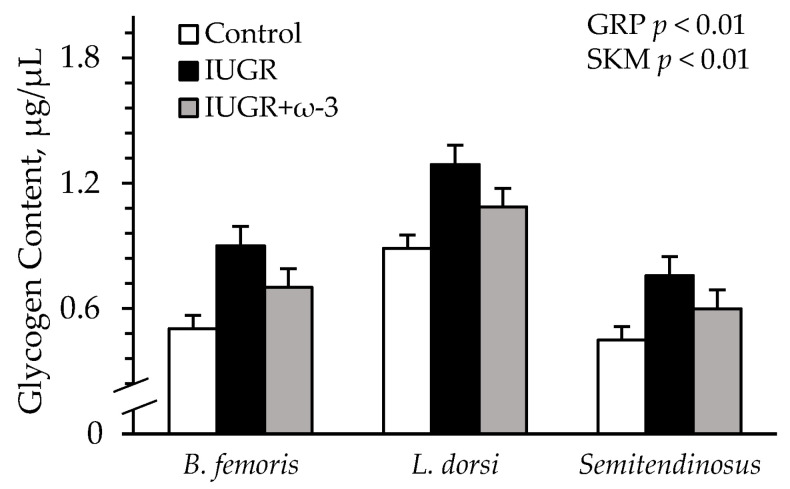
Skeletal muscle glycogen content in IUGR-born neonatal lambs administered daily oral ω-3 PUFA supplements. Glycogen was assessed in *biceps femoris*, *longissimus dorsi*, and *semitendinosus* from control (n = 12), IUGR (n = 11), and IUGR+ω-3 lambs (n = 11). Effects of experimental group (GRP), muscle (SKM), and their interaction were evaluated and noted where significant (*p* < 0.05).

Representative immunoblot images are shown in respective figures, and whole gels are shown in Figure S3. *Semitendinosus* TNFR1 was greater (*p* < 0.05) for IUGR and IUGR+ω3 lambs than for controls (Figure 8A). IL6R was greater (*p* < 0.05) for IUGR lambs but not IUGR+ω3 lambs than for controls (Figure 8B). TLR4 did not differ among groups (Figure 8C). β_2_-AR was less (*p* < 0.05) for IUGR lambs than for controls and was least (*p* < 0.05) for IUGR+ω3 lambs (Figure 8D). No experimental group x muscle interaction was observed for muscle glycogen content. Glycogen was greater (*p* < 0.05) for IUGR lambs than controls and was intermediate for IUGR+ω3 lambs (Figure 9). Glycogen content was also greater (*p* < 0.05) for the *longissimus dorsi* than the *semitendinosus* or *biceps femoris*.

### 3.7. Effects of Sex and Birth Number

For all parameters, the observed effects of sex are summarized in Table S1 and the effects of birth number are summarized in Table S2.

## 4. Discussion

In this study, we found that IUGR-born lambs exhibited substantially heightened inflammatory tone as neonates and that ameliorating it improved deficits in metabolic health. Greater circulating cytokines and enhanced inflammatory signaling pathways in skeletal muscle were previously observed in IUGR fetuses, a result of the chronic fetal hypoxia present in late gestation [15,16,40,41]. The postnatal persistence of inflammation in the present study indicated that it is not just a transient fetal response to hypoxia but a product of fetal programming. Early postnatal supplementation of ω-3 PUFA to IUGR-born lambs improved or eliminated multiple components of this heightened inflammatory tone. In turn, deficits in muscle glucose metabolism and insulin responsiveness improved, and the apparent compensatory increase in non-glucose substrate oxidation (presumably fatty acids) returned to normal. These improvements establish inflammatory programming as a key underlying factor in skeletal muscle metabolic dysfunction following fetal stress. Inflammatory mitigation also improved poor pancreatic β cell stimulus-secretion coupling, a known consequence of IUGR programming [18,31,33], but had little impact on the lipid profiles of IUGR-born lambs. The effects of ω-3 PUFA supplementation occurred without an increase in circulating EPA or DHA concentrations, which was not unexpected. Although reduced by IUGR at birth, postnatal blood EPA concentrations are lowly correlated with dietary ω-3 PUFA intake due to rapid clearance into plasma membranes [42,43,44]. Regardless, improved metabolic and cardiovascular indicators following ω-3 PUFA supplementation establish inflammatory programming as an effective mechanistic target for postnatal intervention addressing IUGR outcomes.

The large deficits in glucose oxidative metabolism by IUGR skeletal muscle were particularly responsive to the mitigation of inflammatory tone. Primary muscle isolated from the hamstring of IUGR-born lambs oxidized glucose at rates that were about 30% less than normal, despite a rise in total oxidative metabolism indicated by greater O_2_ consumption rates. Under hyperinsulinemia, IUGR-born lambs exhibited similarly diminished glucose oxidation across the tissues of the hindlimb, which are about two-thirds skeletal muscle [45]. The reduced oxidation of glucose was not a result of its intracellular availability, as in vivo and ex vivo glucose uptake by IUGR muscle was normal. Rather, the utilization of glucose was shifted to storage and anaerobic glycolysis. The intracellular glycogen content of the loin and hamstring muscles was 45–80% greater in IUGR-born lambs, and their hindlimb tissues produced and secreted about twice the amount of lactate. These metabolic patterns are consistent with impaired TCA cycle and Electron Transport Chain components [7,46,47] and preferential oxidation of fatty acids [7,8] observed in IUGR fetal muscle. Reduced β2 adrenergic signaling in muscle and fat was previously identified as a contributing factor to IUGR metabolic dysfunction [17,18,31,33,48]. However, chronic fetal inflammation produces a comparable IUGR metabolic phenotype [13,19,25,49,50]. Moreover, metabolic improvements by daily β2 adrenergic stimulation were concurrent with the resolution of systemic inflammation in heat stress-induced IUGR-born lambs [17,18]. In most tissues, inflammatory cytokines increase glucose oxidation when exposure time is brief [24,51,52,53] but reduce it following exposure lasting more than a few hours [54,55,56,57,58,59,60]. Additionally, cytokines markedly increase intramuscular glycogen content by inactivating glycogenolytic enzymes [61,62] and increasing glycogen synthesis [51,63,64,65]. They likewise induce anaerobic lactate production [52,66], which further pulls glucose away from oxidative metabolism.

Greater O_2_ consumption by IUGR muscle indicates that poor glucose oxidation was offset by the compensatory oxidation of non-glucose substrates. Although amino acid oxidation remains normal [67,68,69,70], β oxidation pathways are enhanced in IUGR fetal muscle [8,71], resulting in greater fatty acid oxidation that persists postnatal [72,73,74]. Normal O_2_ consumption rates following ω-3 PUFA supplementation may illustrate an underlying role for inflammation in compensatory fatty acid oxidation. Studies in humans and rodents show that the exposure of uncompromised muscle to cytokines, particularly interleukins, markedly increased fatty acid oxidation via PPARα and JAK-STAT pathways [63,75,76,77], independent of lipid uptake and esterification rates [75,78]. Although the canonical inflammatory mediator TLR4 paradoxically increases glucose oxidation and reduces fatty acid oxidation [79,80], its expression in muscle was not enhanced by IUGR in the present study. The metabolic improvements created by inflammatory mitigation were not associated with improvements in impaired β2 adrenergic tone, which is a hallmark of IUGR skeletal muscle [15,17,33]. Inflammatory and adrenergic regulation of muscle are often inversely linked [81,82], and prolonged cytokine exposure desensitizes β2 adrenergic tone by uncoupling the adrenoceptor from adenylyl cyclase [83,84,85]. Although β2 adrenoceptor deficits were resolved by ω-3 PUFA infusion in IUGR fetal sheep [15], they were unexpectedly worsened further by oral ω-3 PUFA supplementation in our IUGR-born neonates. The mechanism for this is unclear, but β2 adrenoceptor negative feedback loops are facilitated by ERK1/2, which can also be activated by ω-3 PUFA [26,81,82].

Pancreatic β cell function improved when heightened inflammatory tone was alleviated in IUGR-born lambs. The large reduction in glucose-stimulated insulin secretion exhibited by IUGR lambs despite normal insulin secretion at euglycemia is a hallmark of IUGR fetuses that results from impaired β cell glucose oxidation [86,87,88]. Fetal hypoxia is the primary catalyst for β cell dysfunction, and resolving it rescues normal insulin secretion in the IUGR fetus and offspring [31,88,89]. Hypoxia-induced hypercatecholaminemia is a well-established inhibitor of fetal insulin stimulus-secretion coupling [90,91]. In fact, catecholamine infusion into otherwise uncompromised fetuses essentially abolished glucose-stimulated insulin secretion [92,93,94]. However, adrenergic mechanisms likely do not explain programmed β cell dysfunction, as glucose-stimulated insulin secretion was not only recovered but enhanced following the discontinuation of catecholamine infusions [92,93,94]. Moreover, preventing the catecholamine response via adrenal demedullation only partially recovered glucose-stimulated insulin secretion in IUGR and experimentally hypoxic fetuses [89,90]. Inflammatory cytokines, which are also secreted in response to hypoxia, likewise impair insulin production and secretion [95,96,97]. Although improved glucose-stimulated insulin secretion following inflammatory mitigation indicates inflammatory programming in IUGR β cells, fetal inflammation alone may not produce permanent β cell dysfunction [19]. Since β blockers reduce endotoxin-induced islet inflammation, it is possible that inflammatory programing in β cells requires concurrent adrenergic exposure [98].

Hypertension was exhibited by IUGR-born lambs but was improved by the reduction in systemic inflammation. High blood pressure following IUGR is associated with impaired nitric oxide synthase, which limits the capacity for vasodilation [99,100]. Nitric oxide synthase activity can be disrupted by high circulating concentrations of inflammatory cytokines, catecholamines, and K^+^ [101,102], all of which occur with IUGR. Although circulating catecholamines were not assessed in the present study, inflammatory cytokines and K^+^ were returned to normal by ω-3 PUFA supplementation, which is consistent with ω-3 PUFA actions on eNOS-facilitated vasodilation [103]. Conversely, diminished erythrocyte populations and hematology metrics were not recovered when inflammatory tone was mitigated. This was somewhat surprising, as fewer circulating red blood cells with greater volume were previously found to be associated with systemic inflammation [104,105,106]. Erythrocyte dysregulation may have resulted from other IUGR conditions, including dyslipidemia and oxidative stress [105]. Importantly, daily supplementation of ω-3 PUFA resulted in only one apparent off-target effect, a modest increase in blood urea nitrogen despite no effect of IUGR.

## 5. Conclusions

The findings of this study demonstrate a clear role for inflammatory programming in the metabolic deficits that characterize IUGR-born offspring. Early postnatal mitigation of heightened inflammatory tone by the oral supplementation of anti-inflammatory ω-3 PUFA consequently improved the metabolic health of lambs made IUGR by maternal heat stress. The greatest improvements were associated with skeletal muscle glucose metabolism, glucose-stimulated insulin secretion, and arterial blood pressure, all of which are known to be responsive to inflammatory regulation even under normal conditions. Inflammatory mitigation had surprisingly little impact on lipid flux, although the effects of IUGR on circulating lipids were quite modest compared to other metabolic parameters. Together, these findings demonstrate that heightened inflammatory tone is an effective target for postnatal supplemental therapies to improve metabolic outcomes following heat stress-induced IUGR.

## Data Availability

The datasets generated in this study are not publicly available but may be made available upon reasonable request to the corresponding author.

## Supplementary Materials

The following supporting information can be downloaded at https://www.mdpi.com/article/10.3390/metabo15060346/s1, Figure S1. Blood gases and associated components under hyperglycemia in IUGR-born neonatal lambs administered daily oral ω-3 PUFA supplements. Figure S2. Blood gases and associated components under hyperinsulinemia in IUGR-born neonatal lambs administered daily oral ω-3 PUFA supplements. Figure S3. Expression of inflammatory and adrenergic proteins in IUGR-born neonatal lambs administered daily oral ω-3 PUFA supplements. Table S1. Effects of sex (male/female) on the assessed physiological parameters. Table S2. Effects of birth number (singleton/twin/triplet) on the assessed physiological parameters.

## References

[1] Barker D.J., Eriksson J.G., Forsen T., Osmond C. (2002). Fetal origins of adult disease: Strength of effects and biological basis. Int. J. Epidemiol..

[2] Barker D.J., Hales C.N., Fall C.H., Osmond C., Phipps K., Clark P.M. (1993). Type 2 (non-insulin-dependent) diabetes mellitus, hypertension and hyperlipidaemia (syndrome X): Relation to reduced fetal growth. Diabetologia.

[3] White M.R., Yates D.T. (2023). Dousing the flame: Reviewing the mechanisms of inflammatory programming during stress-induced intrauterine growth restriction and the potential for ω-3 polyunsaturated fatty acid intervention. Front. Physiol..

[4] Gibbs R.L., Yates D.T. (2021). The Price of Surviving on Adrenaline: Developmental Programming Responses to Chronic Fetal Hypercatecholaminemia Contribute to Poor Muscle Growth Capacity and Metabolic Dysfunction in IUGR-Born Offspring. Front. Anim. Sci..

[5] Limesand S.W., Rozance P.J., Macko A.R., Anderson M.J., Kelly A.C., Hay W.W. (2013). Reductions in insulin concentrations and beta-cell mass precede growth restriction in sheep fetuses with placental insufficiency. Am. J. Physiol. Endocrinol. Metab..

[6] Macko A.R., Yates D.T., Chen X., Green A.S., Kelly A.C., Brown L.D., Limesand S.W. (2013). Elevated plasma norepinephrine inhibits insulin secretion, but adrenergic blockade reveals enhanced beta-cell responsiveness in an ovine model of placental insufficiency at 0.7 of gestation. J. Dev. Orig. Health Dis..

[7] Pendleton A.L., Antolic A.T., Kelly A.C., Davis M.A., Camacho L.E., Doubleday K., Anderson M.J., Langlais P.R., Lynch R.M., Limesand S.W. (2020). Lower oxygen consumption and Complex I activity in mitochondria isolated from skeletal muscle of fetal sheep with intrauterine growth restriction. Am. J. Physiol. Endocrinol. Metab..

[8] Zhao W., Kelly A.C., Luna-Ramirez R.I., Bidwell C.A., Anderson M.J., Limesand S.W. (2023). Decreased Pyruvate but not Fatty Acid Driven Mitochondrial Respiration in Skeletal Muscle of Growth Restricted Fetal Sheep. Int. J. Mol. Sci..

[9] Jones C.T., Ritchie J.W. (1978). The metabolic and endocrine effects of circulating catecholamines in fetal sheep. J. Physiol..

[10] Jones C.T., Robinson J.S. (1983). Studies on experimental growth retardation in sheep. Plasma catecholamines in fetuses with small placenta. J. Dev. Physiol..

[11] Jones C.T., Ritchie J.W., Walker D. (1983). The effects of hypoxia on glucose turnover in the fetal sheep. J. Dev. Physiol..

[12] Chang E.I., Zárate M.A., Rabaglino M.B., Richards E.M., Keller-Wood M., Wood C.E. (2016). Ketamine suppresses hypoxia-induced inflammatory responses in the late-gestation ovine fetal kidney cortex. J. Physiol..

[13] Cadaret C.N., Merrick E.M., Barnes T.L., Beede K.A., Posont R.J., Petersen J.L., Yates D.T. (2019). Sustained maternal inflammation during the early third-trimester yields intrauterine growth restriction, impaired skeletal muscle glucose metabolism, and diminished beta-cell function in fetal sheep. J. Anim. Sci..

[14] Zarate M.A., Chang E.I., Wood C.E. (2018). Effects of ketamine on the fetal transcriptomic response to umbilical cord occlusion: Comparison with hypoxic hypoxia in the cerebral cortex. J. Physiol..

[15] Beer H.N., Lacey T.A., Gibbs R.L., Most M.S., Hicks Z.M., Grijalva P.C., Marks-Nelson E.S., Schmidt T.B., Petersen J.L., Yates D.T. (2024). Daily Eicosapentaenoic Acid Infusion in IUGR Fetal Lambs Reduced Systemic Inflammation, Increased Muscle ADRβ2 Content, and Improved Myoblast Function and Muscle Growth. Metabolites.

[16] Posont R.J., Most M.S., Cadaret C.N., Marks-Nelson E.S., Beede K.A., Limesand S.W., Schmidt T.B., Petersen J.L., Yates D.T. (2022). Primary myoblasts from intrauterine growth-restricted fetal sheep exhibit intrinsic dysfunction of proliferation and differentiation that coincides with enrichment of inflammatory cytokine signaling pathways. J. Anim. Sci..

[17] Gibbs R.L., Swanson R.M., Beard J.K., Hicks Z.M., Most M.S., Beer H.N., Grijalva P.C., Clement S.M., Marks-Nelson E.S., Schmidt T.B. (2023). Daily injection of the β2 adrenergic agonist clenbuterol improved poor muscle growth and body composition in lambs following heat stress-induced intrauterine growth restriction. Front. Physiol..

[18] Gibbs R.L., Wilson J.A., Swanson R.M., Beard J.K., Hicks Z.M., Beer H.N., Marks-Nelson E.S., Schmidt T.B., Petersen J.L., Yates D.T. (2024). Daily Injection of the β2 Adrenergic Agonist Clenbuterol Improved Muscle Glucose Metabolism, Glucose-Stimulated Insulin Secretion, and Hyperlipidemia in Juvenile Lambs Following Heat-Stress-Induced Intrauterine Growth Restriction. Metabolites.

[19] Posont R.J., Cadaret C.N., Beard J.K., Swanson R.M., Gibbs R.L., Marks-Nelson E.S., Petersen J.L., Yates D.T. (2021). Maternofetal inflammation induced for two weeks in late gestation reduced birthweight and impaired neonatal growth and skeletal muscle glucose metabolism in lambs. J. Anim. Sci..

[20] Bai G., Chen J., Liu Y., Chen J., Yan H., You J., Zou T. (2024). Neonatal resveratrol administration promotes skeletal muscle growth and insulin sensitivity in intrauterine growth-retarded suckling piglets associated with activation of FGF21-AMPKα pathway. J. Sci. Food Agric..

[21] Laskowska M., Laskowska K., Leszczyńska-Gorzelak B., Oleszczuk J. (2007). Maternal and umbilical sTNF-R1 in preeclamptic pregnancies with intrauterine normal and growth retarded fetus. Hypertens. Pregnancy.

[22] Rotter V., Nagaev I., Smith U. (2003). Interleukin-6 (IL-6) induces insulin resistance in 3T3-L1 adipocytes and is, like IL-8 and tumor necrosis factor-alpha, overexpressed in human fat cells from insulin-resistant subjects. J. Biol. Chem..

[23] Al-Khalili L., Bouzakri K., Glund S., Lonnqvist F., Koistinen H.A., Krook A. (2006). Signaling specificity of interleukin-6 action on glucose and lipid metabolism in skeletal muscle. Mol. Endocrinol..

[24] Cadaret C.N., Beede K.A., Riley H.E., Yates D.T. (2017). Acute exposure of primary rat soleus muscle to zilpaterol HCl (β2 adrenergic agonist), TNFa, or IL-6 in culture increases glucose oxidation rates independent of the impact on insulin signaling or glucose uptake. Cytokine.

[25] Hicks Z.M. (2023). Mid-Gestation Maternofetal Inflammation Impacts Growth, Skeletal Muscle Glucose Metabolism, and Inflammatory Tone in the Ovine Fetus During Late Gestation. Ph.D. Thesis.

[26] Oh D.Y., Talukdar S., Bae E.J., Imamura T., Morinaga H., Fan W., Li P., Lu W.J., Watkins S.M., Olefsky J.M. (2010). GPR120 is an omega-3 fatty acid receptor mediating potent anti-inflammatory and insulin-sensitizing effects. Cell.

[27] Weldon S.M., Mullen A.C., Loscher C.E., Hurley L.A., Roche H.M. (2007). Docosahexaenoic acid induces an anti-inflammatory profile in lipopolysaccharide-stimulated human THP-1 macrophages more effectively than eicosapentaenoic acid. J. Nutr. Biochem..

[28] Block R.C., Dier U., Calderonartero P., Shearer G.C., Kakinami L., Larson M.K., Harris W.S., Georas S., Mousa S.A. (2012). The Effects of EPA+DHA and Aspirin on Inflammatory Cytokines and Angiogenesis Factors. World J. Cardiovasc. Dis..

[29] Grytten E., Laupsa-Borge J., Cetin K., Bohov P., Nordrehaug J.E., Skorve J., Berge R.K., Strand E., Bjørndal B., Nygård O. (2025). Inflammatory markers after supplementation with marine n-3 or plant n-6 PUFAs: A randomized double-blind crossover study. J. Lipid Res..

[30] Lacey T.L., Gibbs R.L., Most M.S., Beer H.N., Hicks Z.M., Grijalva P.C., Petersen J.L., Yates D.T. (2021). Decreased fetal biometrics and impaired β-cell function in IUGR fetal sheep are improved by daily ω-3 PUFA infusion. Transl. Anim. Sci..

[31] Cadaret C.N., Posont R.J., Swanson R.M., Beard J.K., Gibbs R.L., Barnes T.L., Marks-Nelson E.S., Petersen J.L., Yates D.T. (2022). Intermittent maternofetal oxygenation during late gestation improved birthweight, neonatal growth, body symmetry, and muscle metabolism in intrauterine growth-restricted lambs. J. Anim. Sci..

[32] Swanson R.M., Tait R.G., Galles B.M., Duffy E.M., Schmidt T.B., Petersen J.L., Yates D.T. (2020). Heat stress-induced deficits in growth, metabolic efficiency, and cardiovascular function coincided with chronic systemic inflammation and hypercatecholaminemia in ractopamine-supplemented feedlot lambs. J. Anim. Sci..

[33] Yates D.T., Camacho L.E., Kelly A.C., Steyn L.V., Davis M.A., Antolic A.T., Anderson M.J., Goyal R., Allen R.E., Papas K.K. (2019). Postnatal beta2 adrenergic treatment improves insulin sensitivity in lambs with IUGR but not persistent defects in pancreatic islets or skeletal muscle. J. Physiol..

[34] Camacho L.E., Chen X., Hay W.W., Limesand S.W. (2017). Enhanced insulin secretion and insulin sensitivity in young lambs with placental insufficiency-induced intrauterine growth restriction. Am. J. Physiol.-Regul. Integr. Comp. Physiol..

[35] Barnes T.L., Burrack R.M., Schmidt T.B., Petersen J.L., Yates D.T. (2021). Sustained heat stress elevated corneal and body surface temperatures and altered circulating leukocytes and metabolic indicators in wether lambs supplemented with ractopamine or zilpaterol. J. Anim. Sci..

[36] Barnes T.L., Cadaret C.N., Beede K.A., Schmidt T.B., Petersen J.L., Yates D.T. (2019). Hypertrophic muscle growth and metabolic efficiency were impaired by chronic heat stress, improved by zilpaterol supplementation, and not affected by ractopamine supplementation in feedlot lambs. J. Anim. Sci..

[37] Paliogiannis P., Ginesu G.C., Tanda C., Feo C.F., Fancellu A., Fois A.G., Mangoni A.A., Sotgia S., Carru C., Porcu A. (2018). Inflammatory cell indexes as preoperative predictors of hospital stay in open elective thoracic surgery. ANZ J. Surg..

[38] Hu B., Yang X.R., Xu Y., Sun Y.F., Sun C., Guo W., Zhang X., Wang W.M., Qiu S.J., Zhou J. (2014). Systemic immune-inflammation index predicts prognosis of patients after curative resection for hepatocellular carcinoma. Clin. Cancer Res..

[39] Qi Q., Zhuang L., Shen Y., Geng Y., Yu S., Chen H., Liu L., Meng Z., Wang P., Chen Z. (2016). A novel systemic inflammation response index (SIRI) for predicting the survival of patients with pancreatic cancer after chemotherapy. Cancer.

[40] Leslie E., Lopez V., Anti N.A.O., Alvarez R., Kafeero I., Welsh D.G., Romero M., Kaushal S., Johnson C.M., Bosviel R. (2021). Gestational long-term hypoxia induces metabolomic reprogramming and phenotypic transformations in fetal sheep pulmonary arteries. Am. J. Physiol. Lung Cell. Mol. Physiol..

[41] Dong Y., Hou W., Wei J., Weiner C.P. (2009). Chronic hypoxemia absent bacterial infection is one cause of the fetal inflammatory response syndrome (FIRS). Reprod. Sci..

[42] von Schacky C. (2009). Use of red blood cell fatty-acid profiles as biomarkers in cardiac disease. Biomark. Med..

[43] Sun Q., Ma J., Campos H., Hankinson S.E., Hu F.B. (2007). Comparison between plasma and erythrocyte fatty acid content as biomarkers of fatty acid intake in US women. Am. J. Clin. Nutr..

[44] Gerling C.J., Mukai K., Chabowski A., Heigenhauser G.J.F., Holloway G.P., Spriet L.L., Jannas-Vela S. (2019). Incorporation of Omega-3 Fatty Acids into Human Skeletal Muscle Sarcolemmal and Mitochondrial Membranes Following 12 Weeks of Fish Oil Supplementation. Front. Physiol..

[45] Hicks Z.H., Beer H.N., Herrera N.J., Gibbs R.L., Lacey T.A., Grijalva P.C., Most M.S., Yates D.T. (2021). Hindlimb tissue composition shifts between the fetal and juvenile stages in the lamb. Transl. Anim. Sci..

[46] Stremming J., Chang E.I., Knaub L.A., Armstrong M.L., Baker P.R., Wesolowski S.R., Reisdorph N., Reusch J.E.B., Brown L.D. (2022). Lower citrate synthase activity, mitochondrial complex expression, and fewer oxidative myofibers characterize skeletal muscle from growth-restricted fetal sheep. Am. J. Physiol. Regul. Integr. Comp. Physiol..

[47] Selak M.A., Storey B.T., Peterside I., Simmons R.A. (2003). Impaired oxidative phosphorylation in skeletal muscle of intrauterine growth-retarded rats. Am. J. Physiol.-Endocrinol. Metab..

[48] Chen X., Fahy A.L., Green A.S., Anderson M.J., Rhoads R.P., Limesand S.W. (2010). β2-Adrenergic receptor desensitization in perirenal adipose tissue in fetuses and lambs with placental insufficiency-induced intrauterine growth restriction. J. Physiol..

[49] Hicks Z.M., Gibbs R.L., Beer H.N., Grijalva P.C., Most M.S., Yates D.T. (2022). PSVIII-B-18 Sustained Maternofetal Inflammation at mid-Gestation Causes Intrauterine Growth Restriction of the Sheep Fetus That is Characterized by Poor Muscle Mass and Asymmetric Body Composition Near Term. J. Anim. Sci..

[50] Cadaret C.N., Posont R.J., Beede K.A., Riley H.E., Loy J.D., Yates D.T. (2019). Maternal inflammation at midgestation impairs subsequent fetal myoblast function and skeletal muscle growth in rats, resulting in intrauterine growth restriction at term. Transl. Anim. Sci..

[51] Glund S., Deshmukh A., Long Y.C., Moller T., Koistinen H.A., Caidahl K., Zierath J.R., Krook A. (2007). Interleukin-6 directly increases glucose metabolism in resting human skeletal muscle. Diabetes.

[52] Tredget E.E., Yu Y.M., Zhong S., Burini R., Okusawa S., Gelfand J.A., Dinarello C.A., Young V.R., Burke J.F. (1988). Role of interleukin 1 and tumor necrosis factor on energy metabolism in rabbits. Am. J. Physiol..

[53] López-Soriano J., Argilés J.M., López-Soriano F.J. (1993). Effects of tumour necrosis factor-alpha on the enzymatic activities related to glucose metabolism. Biochem. Mol. Biol. Int..

[54] Anil T.M., Dandu A., Harsha K., Singh J., Shree N., Kumar V.S., Lakshmi M.N., Sunil V., Harish C., Balamurali G.V. (2014). A novel 11β-hydroxysteroid dehydrogenase type1 inhibitor CNX-010-49 improves hyperglycemia, lipid profile and reduces body weight in diet induced obese C57B6/J mice with a potential to provide cardio protective benefits. BMC Pharmacol. Toxicol..

[55] Adamska A., Nikołajuk A., Karczewska-Kupczewska M., Kowalska I., Otziomek E., Górska M., Strączkowski M. (2012). Relationships between serum adiponectin and soluble TNF-α receptors and glucose and lipid oxidation in lean and obese subjects. Acta Diabetol..

[56] Tao J., Zhang J., Ling Y., McCall C.E., Liu T.F. (2018). Mitochondrial Sirtuin 4 Resolves Immune Tolerance in Monocytes by Rebalancing Glycolysis and Glucose Oxidation Homeostasis. Front. Immunol..

[57] Taylor D.J. (1990). Interleukin-1 stimulation of fibroblast glycolysis is accompanied by reduced glucose oxidation in the tricarboxylic acid cycle. Biochem. Soc. Trans..

[58] Taylor D.J., Faragher E.B., Evanson J.M. (1992). Inflammatory cytokines stimulate glucose uptake and glycolysis but reduce glucose oxidation in human dermal fibroblasts in vitro. Circ. Shock.

[59] Jani S., Da Eira D., Hadday I., Bikopoulos G., Mohasses A., de Pinho R.A., Ceddia R.B. (2021). Distinct mechanisms involving diacylglycerol, ceramides, and inflammation underlie insulin resistance in oxidative and glycolytic muscles from high fat-fed rats. Sci. Rep..

[60] Ramsay T.G., Blomberg L., Caperna T.J. (2013). Methyl-β-cyclodextrin alters adipokine gene expression and glucose metabolism in swine adipose tissue. Animal.

[61] Alonso-Chamorro M., Nieto-Vazquez I., Montori-Grau M., Gomez-Foix A.M., Fernandez-Veledo S., Lorenzo M. (2011). New emerging role of protein-tyrosine phosphatase 1B in the regulation of glycogen metabolism in basal and TNF-α-induced insulin-resistant conditions in an immortalised muscle cell line isolated from mice. Diabetologia.

[62] Araújo A.M., Arruda S.F. (2024). Ameliorating the impairment of glucose utilization in a high-fat diet-induced obesity model through the consumption of Tucum-do-Cerrado (*Bactris Setosa Mart.*). PLoS ONE.

[63] Jiang L.Q., Duque-Guimaraes D.E., Machado U.F., Zierath J.R., Krook A. (2013). Altered response of skeletal muscle to IL-6 in type 2 diabetic patients. Diabetes.

[64] Gudiksen A., Schwartz C.L., Bertholdt L., Joensen E., Knudsen J.G., Pilegaard H. (2016). Lack of Skeletal Muscle IL-6 Affects Pyruvate Dehydrogenase Activity at Rest and during Prolonged Exercise. PLoS ONE.

[65] Klymenko O., Brecklinghaus T., Dille M., Springer C., de Wendt C., Altenhofen D., Binsch C., Knebel B., Scheller J., Hardt C. (2020). Histone deacetylase 5 regulates interleukin 6 secretion and insulin action in skeletal muscle. Mol. Metab..

[66] Michaeli B., Martinez A., Revelly J.P., Cayeux M.C., Chioléro R.L., Tappy L., Berger M.M. (2012). Effects of endotoxin on lactate metabolism in humans. Crit. Care.

[67] Wai S.G., Rozance P.J., Wesolowski S.R., Hay W.W., Brown L.D. (2018). Prolonged amino acid infusion into intrauterine growth restricted fetal sheep increases leucine oxidation rates. Am. J. Physiol. Endocrinol. Metab..

[68] Ross J.C., Fennessey P.V., Wilkening R.B., Battaglia F.C., Meschia G. (1996). Placental transport and fetal utilization of leucine in a model of fetal growth retardation. Am. J. Physiol..

[69] Rozance P.J., Zastoupil L., Wesolowski S.R., Goldstrohm D.A., Strahan B., Cree-Green M., Sheffield-Moore M., Meschia G., Hay W.W., Wilkening R.B. (2018). Skeletal muscle protein accretion rates and hindlimb growth are reduced in late gestation intrauterine growth-restricted fetal sheep. J. Physiol..

[70] Carver T.D., Quick A.A., Teng C.C., Pike A.W., Fennessey P.V., Hay W.W. (1997). Leucine metabolism in chronically hypoglycemic hypoinsulinemic growth-restricted fetal sheep. Am. J. Physiol..

[71] Lane R.H., Kelley D.E., Ritov V.H., Tsirka A.E., Gruetzmacher E.M. (2001). Altered expression and function of mitochondrial beta-oxidation enzymes in juvenile intrauterine-growth-retarded rat skeletal muscle. Pediatr. Res..

[72] Patel D., Kalhan S. (1992). Glycerol metabolism and triglyceride-fatty acid cycling in the human newborn: Effect of maternal diabetes and intrauterine growth retardation. Pediatr. Res..

[73] Brons C., Lilleore S.K., Astrup A., Vaag A. (2016). Disproportionately increased 24-h energy expenditure and fat oxidation in young men with low birth weight during a high-fat overfeeding challenge. Eur. J. Nutr..

[74] Brons C., Jacobsen S., Hiscock N., White A., Nilsson E., Dunger D., Astrup A., Quistorff B., Vaag A. (2012). Effects of high-fat overfeeding on mitochondrial function, glucose and fat metabolism, and adipokine levels in low-birth-weight subjects. Am. J. Physiol. Endocrinol. Metab..

[75] Bruce C.R., Dyck D.J. (2004). Cytokine regulation of skeletal muscle fatty acid metabolism: Effect of interleukin-6 and tumor necrosis factor-alpha. Am. J. Physiol. Endocrinol. Metab..

[76] Pedersen L., Olsen C.H., Pedersen B.K., Hojman P. (2012). Muscle-derived expression of the chemokine CXCL1 attenuates diet-induced obesity and improves fatty acid oxidation in the muscle. Am. J. Physiol. Endocrinol. Metab..

[77] Wolsk E., Mygind H., Grøndahl T.S., Pedersen B.K., van Hall G. (2010). IL-6 selectively stimulates fat metabolism in human skeletal muscle. Am. J. Physiol. Endocrinol. Metab..

[78] Knudsen N.H., Stanya K.J., Hyde A.L., Chalom M.M., Alexander R.K., Liou Y.H., Starost K.A., Gangl M.R., Jacobi D., Liu S. (2020). Interleukin-13 drives metabolic conditioning of muscle to endurance exercise. Science.

[79] Frisard M.I., McMillan R.P., Marchand J., Wahlberg K.A., Wu Y., Voelker K.A., Heilbronn L., Haynie K., Muoio B., Li L. (2010). Toll-like receptor 4 modulates skeletal muscle substrate metabolism. Am. J. Physiol. Endocrinol. Metab..

[80] Frisard M.I., Wu Y., McMillan R.P., Voelker K.A., Wahlberg K.A., Anderson A.S., Boutagy N., Resendes K., Ravussin E., Hulver M.W. (2015). Low levels of lipopolysaccharide modulate mitochondrial oxygen consumption in skeletal muscle. Metabolism.

[81] Kolmus K., Tavernier J., Gerlo S. (2015). β2-Adrenergic receptors in immunity and inflammation: Stressing NF-κB. Brain Behav. Immun..

[82] Lorton D., Bellinger D.L. (2015). Molecular Mechanisms Underlying β-Adrenergic Receptor-Mediated Cross-Talk between Sympathetic Neurons and Immune Cells. Int. J. Mol. Sci..

[83] Chung M.K., Gulick T.S., Rotondo R.E., Schreiner G.F., Lange L.G. (1990). Mechanism of cytokine inhibition of beta-adrenergic agonist stimulation of cyclic AMP in rat cardiac myocytes. Impairment of signal transduction. Circ. Res..

[84] Gulick T., Chung M.K., Pieper S.J., Lange L.G., Schreiner G.F. (1989). Interleukin 1 and tumor necrosis factor inhibit cardiac myocyte beta-adrenergic responsiveness. Proc. Natl. Acad. Sci. USA.

[85] Bick R.J., Liao J.P., King T.W., LeMaistre A., McMillin J.B., Buja L.M. (1997). Temporal effects of cytokines on neonatal cardiac myocyte Ca2+ transients and adenylate cyclase activity. Am. J. Physiol..

[86] Leos R.A., Anderson M.J., Chen X., Pugmire J., Anderson K.A., Limesand S.W. (2010). Chronic exposure to elevated norepinephrine suppresses insulin secretion in fetal sheep with placental insufficiency and intrauterine growth restriction. Am. J. Physiol. Endocrinol. Metab..

[87] Limesand S.W., Rozance P.J., Zerbe G.O., Hutton J.C., Hay W.W. (2006). Attenuated insulin release and storage in fetal sheep pancreatic islets with intrauterine growth restriction. Endocrinology.

[88] Camacho L.E., Davis M.A., Kelly A.C., Steffens N.R., Anderson M.J., Limesand S.W. (2022). Prenatal Oxygen and Glucose Therapy Normalizes Insulin Secretion and Action in Growth-Restricted Fetal Sheep. Endocrinology.

[89] Macko A.R., Yates D.T., Chen X., Shelton L.A., Kelly A.C., Davis M.A., Camacho L.E., Anderson M.J., Limesand S.W. (2016). Adrenal Demedullation and Oxygen Supplementation Independently Increase Glucose-Stimulated Insulin Concentrations in Fetal Sheep With Intrauterine Growth Restriction. Endocrinology.

[90] Yates D.T., Macko A.R., Chen X., Green A.S., Kelly A.C., Anderson M.J., Fowden A.L., Limesand S.W. (2012). Hypoxaemia-induced catecholamine secretion from adrenal chromaffin cells inhibits glucose-stimulated hyperinsulinaemia in fetal sheep. J. Physiol..

[91] Benjamin J.S., Culpepper C.B., Brown L.D., Wesolowski S.R., Jonker S.S., Davis M.A., Limesand S.W., Wilkening R.B., Hay W.W., Rozance P.J. (2017). Chronic anemic hypoxemia attenuates glucose-stimulated insulin secretion in fetal sheep. Am. J. Physiol. Regul. Integr. Comp. Physiol..

[92] Chen X., Green A.S., Macko A.R., Yates D.T., Kelly A.C., Limesand S.W. (2014). Enhanced insulin secretion responsiveness and islet adrenergic desensitization after chronic norepinephrine suppression is discontinued in fetal sheep. Am. J. Physiol. Endocrinol. Metab..

[93] Chen X., Kelly A.C., Yates D.T., Macko A.R., Lynch R.M., Limesand S.W. (2017). Islet adaptations in fetal sheep persist following chronic exposure to high norepinephrine. J. Endocrinol..

[94] Li R., Huang H., Limesand S.W., Chen X. (2021). Pancreatic Islets Exhibit Dysregulated Adaptation of Insulin Secretion after Chronic Epinephrine Exposure. Curr. Issues Mol. Biol..

[95] Hadjivassiliou V., Green M.H., James R.F., Swift S.M., Clayton H.A., Green I.C. (1998). Insulin secretion, DNA damage, and apoptosis in human and rat islets of Langerhans following exposure to nitric oxide, peroxynitrite, and cytokines. Nitric Oxide.

[96] Zhang Y., Jin W., Zhang D., Lin C., He H., Xie F., Gan L., Fu W., Wu L., Wu Y. (2022). TNF-α Antagonizes the Effect of Leptin on Insulin Secretion through FOXO1-Dependent Transcriptional Suppression of LepRb in INS-1 Cells. Oxid. Med. Cell. Longev..

[97] Eizirik D.L., Mandrup-Poulsen T. (2001). A choice of death--the signal-transduction of immune-mediated beta-cell apoptosis. Diabetologia.

[98] Amirshahrokhi K., Zohouri A. (2021). Carvedilol prevents pancreatic β-cell damage and the development of type 1 diabetes in mice by the inhibition of proinflammatory cytokines, NF-κB, COX-2, iNOS and oxidative stress. Cytokine.

[99] Franco M.d.C.P., Arruda R.M.M.P., Dantas A.P.V., Kawamoto E.M., Fortes Z.B., Scavone C., Carvalho M.H.C., Tostes R.C.A., Nigro D. (2002). Intrauterine undernutrition: Expression and activity of the endothelial nitric oxide synthase in male and female adult offspring. Cardiovasc. Res..

[100] Grandvuillemin I., Buffat C., Boubred F., Lamy E., Fromonot J., Charpiot P., Simoncini S., Sabatier F., Dignat-George F., Peyter A.C. (2018). Arginase upregulation and eNOS uncoupling contribute to impaired endothelium-dependent vasodilation in a rat model of intrauterine growth restriction. Am. J. Physiol. Regul. Integr. Comp. Physiol..

[101] Dinh Q.N., Drummond G.R., Sobey C.G., Chrissobolis S. (2014). Roles of inflammation, oxidative stress, and vascular dysfunction in hypertension. Biomed. Res. Int..

[102] Kalinowski L., Dobrucki L.W., Szczepanska-Konkel M., Jankowski M., Martyniec L., Angielski S., Malinski T. (2003). Third-generation beta-blockers stimulate nitric oxide release from endothelial cells through ATP efflux: A novel mechanism for antihypertensive action. Circulation.

[103] Bercea C.I., Cottrell G.S., Tamagnini F., McNeish A.J. (2021). Omega-3 polyunsaturated fatty acids and hypertension: A review of vasodilatory mechanisms of docosahexaenoic acid and eicosapentaenoic acid. Br. J. Pharmacol..

[104] Lippi G., Targher G., Montagnana M., Salvagno G.L., Zoppini G., Guidi G.C. (2009). Relation Between Red Blood Cell Distribution Width and Inflammatory Biomarkers in a Large Cohort of Unselected Outpatients. Arch. Pathol. Lab. Med..

[105] Salvagno G.L., Sanchis-Gomar F., Picanza A., Lippi G. (2015). Red blood cell distribution width: A simple parameter with multiple clinical applications. Crit. Rev. Clin. Lab. Sci..

[106] Rondanelli M., Perna S., Alalwan T.A., Cazzola R., Gasparri C., Infantino V., Perdoni F., Iannello G., Pepe D., Guido D. (2020). A structural equation model to assess the pathways of body adiposity and inflammation status on dysmetabolic biomarkers via red cell distribution width and mean corpuscular volume: A cross-sectional study in overweight and obese subjects. Lipids Health Dis..

