# Supplementary material for: Reducing Systemic Inflammation in IUGR-Born Neonatal Lambs via Daily Oral ω-3 PUFA Supplement Improved Skeletal Muscle Glucose Metabolism, Glucose-Stimulated Insulin Secretion, and Blood Pressure
Source: Metabolites. 2025 May 22;15(6):346. doi: 10.3390/metabo15060346 (PMC12195305; doi:10.3390/metabo15060346)
Supplement: Supplementary file 1 [file metabolites-15-00346-s001.zip › metabolites-3637807-supplementary.pdf]

Table S1. Effects of sex (male/female) on the assessed physiological parameters in neonatal lambs.

Variable	Female	Male	*p*-value
Weekly Blood Samples			
Ca^{2+}	1.35 ± 0.01x	1.38 ± 0.01y	0.06
MPV	5.03 ± 0.02a	4.96 ± 0.02b	0.04
Systolic BP	120 ± 3a	131 ± 3b	<0.01
GSIS			
Glc-to-Insulin	3.53 ± 0.25a	2.59 ± 0.26b	<0.01
pH	7.439 ± 0.003a	7.425 ± 0.003b	<0.01
pCO_2	43.58 ± 0.27a	41.50 ± 0.29b	<0.01
HCO_3^{2-}	28.6 ± 0.2a	27.01 ± 0.24b	<0.01
Base Excess	4.5 ± 0.2a	3.1 ± 0.3b	<0.01
Cl^-	107.7 ± 0.6a	110.8 ± 0.6b	<0.01
Ca^{2+}	1.39 ± 0.01a	1.44 ± 0.01b	<0.01
BUN	11.1 ± 0.2a	8.7 ± 0.2b	<0.01
HEC			
Blood Flow	60 ± 4a	76 ± 4b	<0.01
Insulin	13.3 ± 0.7a	15.5 ± 0.7b	0.03
pCO_2	42.7 ± 0.2a	42.0 ± 0.3b	0.03
Hemoglobin	10.3 ± 0.1a	10.6 ± 0.1b	0.02
Hematocrit	31.7 ± 0.2a	32.5 ± 0.3b	0.02
BUN	11.5 ± 0.3a	10.5 ± 0.2b	0.01
Triglycerides	39.9 ± 2.1a	31.2 ± 2.1b	<0.01

Table S2. Effects of birth number (singleton/twin/triplet) on the assessed physiological parameters in neonatal lambs.

Variable	Singleton	Twins	Triplets	*p*-value
Weekly Blood Samples				
pH	7.393 ± 0.006a	7.418 ± 0.004b	7.413 ± 0.007b	0.01
HCO_3^{2-}	29.41 ± 0.37a	31.27 ± 0.29b	30.17 ± 0.43a	<0.01
Base Excess	4.62 ± 0.40a	6.56 ± 0.31b	5.58 ± 0.47c	<0.01
Hemoglobin	12.28 ± 0.17a	11.97 ± 0.13a	11.13 ± 0.19b	<0.01
Hematocrit	37.63 ± 0.53a	36.65 ± 0.41a	33.99 ± 0.58b	<0.01
CO-Hb	1.97 ± 0.17a	2.23 ± 0.13a	2.65 ± 0.20b	0.04
Cl^-	117.8 ± 0.8a	114.8 ± 0.6b	117.2 ± 0.9a	0.01
Total WBC	6.72 ± 0.26a	5.91 ± 0.21b	5.77 ± 0.31b	0.03
Lymphocytes	3.21 ± 0.11a	2.74 ± 0.09b	2.94 ± 0.13b	<0.01
Granulocytes	3.04 ± 0.15a	2.54 ± 0.12b	2.26 ± 0.18b	<0.01
Red Blood Cells	8.9 ± 0.1a	8.7 ± 0.1a	8.4 ± 0.1b	0.02
Systolic BP	129 ± 3a	131 ± 4a	118 ± 4b	0.01
GSIS				
Insulin	10.8 ± 1.0a	8.0 ± 0.9b	6.4 ± 1.3b	0.03
Glucose	10.3 ± 0.1a	10.6 ± 0.1b	11.0 ± 0.2c	<0.01
pH	7.428 ± 0.004a	7.442 ± 0.003b	7.424 ± 0.005a	<0.01
pCO_2	41.8 ± 0.3a	42.3 ± 0.3a	43.7 ± 0.4b	<0.01
HCO_3^{2-}	27.2 ± 0.3a	28.5 ± 0.2b	27.8 ± 0.4a	<0.01
Base Excess	3.2 ± 0.3a	4.7 ± 0.2b	3.5 ± 0.4a	<0.01
Hemoglobin	11.3 ± 0.1a	10.7 ± 0.1b	10.6 ± 0.2b	<0.01
Hematocrit	35.9 ± 1.6a	34.7 ± 1.6b	34.0 ± 1.7b	<0.01
O_2-Hb	91.1 ± 0.4a	92.2 ± 0.3b	89.4 ± 0.6c	<0.01
Na^+	148.7 ± 0.6a	146.0 ± 0.5b	149.8 ± 0.8a	<0.01
Cl^-	110.2 ± 0.7a	107.0 ± 0.5b	110.9 ± 0.9a	<0.01
pO_2	76.3 ± 0.9a	76.7 ± 0.7a	69.1 ± 1.2b	<0.01
NEFA	0.8 ± 0.1a	0.7 ± 0.1a	1.1 ± 0.1b	<0.01
BUN	10.0 ± 0.2a	9.3 ± 0.2b	10.7 ± 0.3c	<0.01
HDL-C	39.3 ± 0.9a	42.7 ± 0.7b	40.9 ± 1.1ab	<0.01
HEC				
Blood Flow	68 ± 4a	61 ± 4b	75 ± 5c	<0.01
Glucose	6.47 ± 0.09a	6.59 ± 0.07a	6.13 ± 0.10b	<0.01
Lactate	0.60 ± 0.02a	0.52 ± 0.02b	0.50 ± 0.03b	<0.01
pH	7.43 ± 0.001a	7.44 ± 0.001b	7.45 ± 0.001c	<0.01
HCO_3^{2-}	27.95 ± 0.26a	28.45 ± 0.21b	28.96 ± 0.30c	0.02
Base Excess	4.00 ± 0.27a	4.47 ± 0.22b	5.08 ± 0.30c	0.01
Hemoglobin	10.84 ± 0.10a	10.41 ± 0.07b	10.15 ± 0.12c	<0.01
Hematocrit	33.23 ± 0.30a	31.92 ± 0.22b	31.10 ± 0.36c	<0.01
O_2-Hb	91.87 ± 0.29a	92.43 ± 0.21a	94.05 ± 0.35b	<0.01
CO-Hb	3.37 ± 0.19a	3.11 ± 0.14a	5.21 ± 0.22b	<0.01
K^+	4.10 ± 0.04a	3.95 ± 0.03b	4.07 ± 0.05a	0.02
Ca^{2+}	1.43 ± 0.01a	1.39 ± 0.01b	1.36 ± 0.01c	<0.01
NEFA	0.85 ± 0.08a	0.67 ± 0.06b	0.99 ± 0.10a	0.01
Triglycerides	38.5 ± 2.5a	29.3 ± 1.8b	44.1 ± 3.3a	<0.01
OCR	45.19 ± 11.81a	57.48 ± 6.12a	96.23 ± 11.52b	0.01

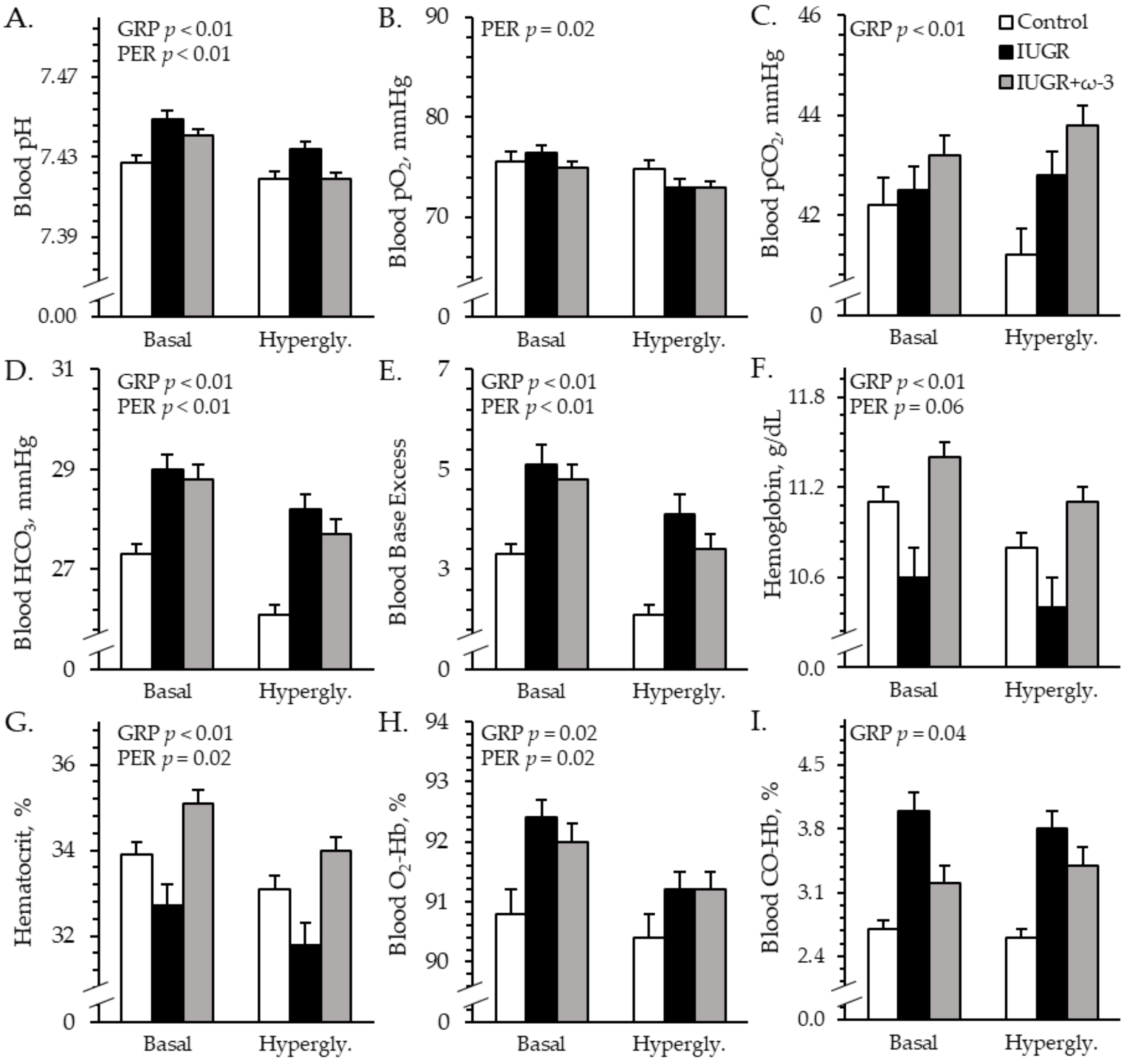

Figure S1. Blood gases and associated components under hyperglycemia in IUGR-born neonatal lambs administered daily oral ω-3 PUFA supplements. Blood was sampled under basal conditions and during a square-wave hyperglycemic clamp in control (n = 12), IUGR (n = 11), and IUGR+ω-3 lambs (n = 11). Data are shown for blood pH (A.), partial pressure of O_2 (B.), partial pressure of CO_2 (C.), HCO_3^{2-} (D.), base excess (E.), hemoglobin (F.), hematocrit (G.), oxyhemoglobin (H.), and carboxyhemoglobin (I.). Effects of experimental group (GRP), period (PER), and the interaction were evaluated and are noted where significant ($p < 0.05$).

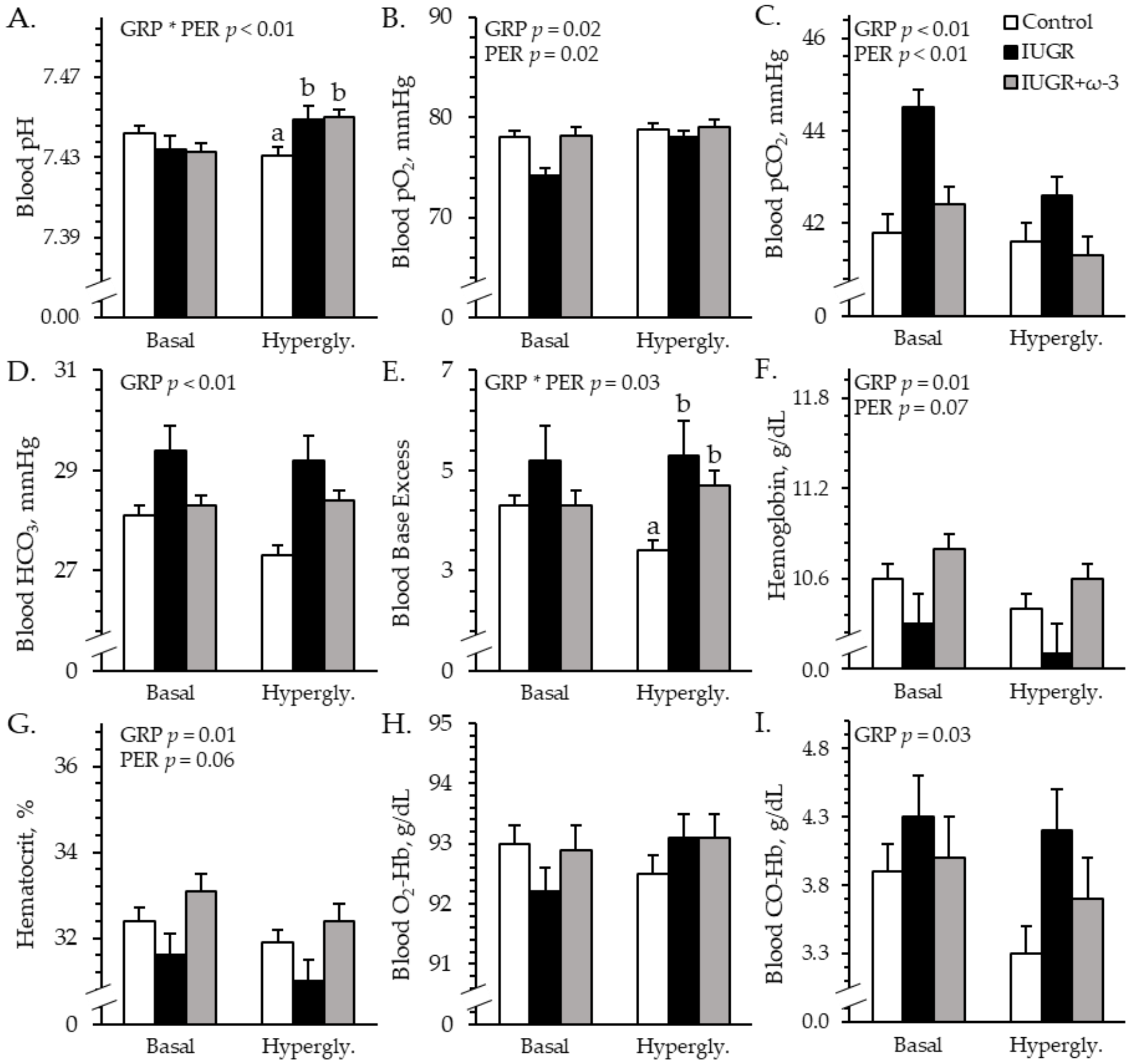

Figure S2. Blood gases and associated components under hyperinsulinemia in IUGR-born neonatal lambs administered daily oral ω-3 PUFA supplements. Blood was sampled under basal conditions and during a hyperinsulinemic-euglycemic clamp (HEC) in control (n = 12), IUGR (n = 11), and IUGR+ω-3 lambs (n = 11). Data are shown for blood pH (A.), partial pressure of O_2 (B.), partial pressure of CO_2 (C.), HCO_3^{2-} (D.), base excess (E.), hemoglobin (F.), hematocrit (G.), oxyhemoglobin (H.), and carboxyhemoglobin (I.). Effects of experimental group (GRP), period (PER), and the interaction were evaluated and are noted where significant ($p < 0.05$). [a, b, c] Means with different superscripts differ ($p < 0.05$).

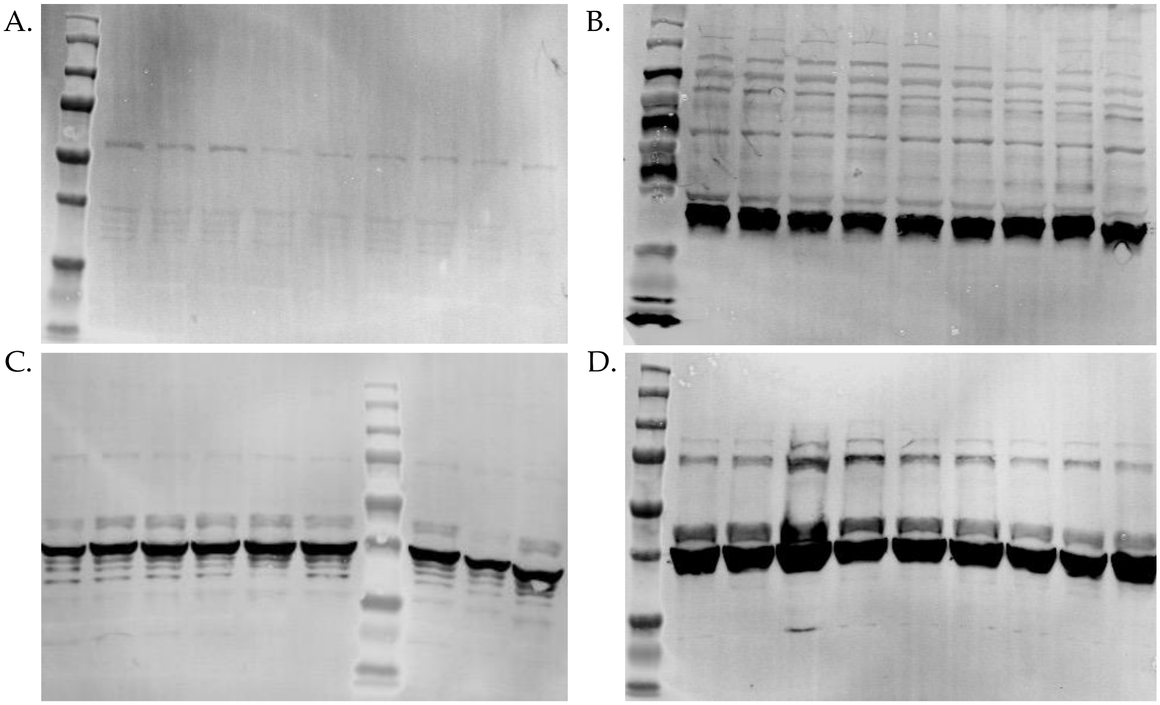

Figure S3. Expression of inflammatory and adrenergic proteins in IUGR-born neonatal lambs administered daily oral ω-3 PUFA supplements. *Semitendinosus* muscle was collected and frozen at necropsy for control (n = 12), IUGR (n = 11), and IUGR+ω-3 lambs (n = 11). Data are shown for β2-AR (A.), TLR4 (B.), TNFR1 (C.), and IL6R (D.).
